# A Bayesian Model of Category-Specific Emotional Brain Responses

**DOI:** 10.1371/journal.pcbi.1004066

**Published:** 2015-04-08

**Authors:** Tor D. Wager, Jian Kang, Timothy D. Johnson, Thomas E. Nichols, Ajay B. Satpute, Lisa Feldman Barrett

**Affiliations:** 1 Department of Psychology and Neuroscience and the Institute for Cognitive Science, University of Colorado, Boulder, Colorado, United States of America; 2 Department of Biostatistics and Bioinformatics, Department of Radiology and Imaging Sciences, Emory University, Atlanta, Georgia, United States of America; 3 Department of Biostatistics, University of Michigan, Ann Arbor, Michigan, United States of America; 4 Department of Statistics and Warwick Manufacturing Group, University of Warwick, Coventry, United Kingdom; 5 Functional Magnetic Resonance Imaging of the Brain (FMRIB) Centre, Nuffield Department of Clinical Neurosciences, University of Oxford, Oxford, United Kingdom; 6 Department of Psychology, Northeastern University, Boston, Massachusetts, United States of America; 7 Massachusetts General Hospital/Harvard Medical School, Boston, Massachusetts, United States of America; Institute of Cognitive Neuroscience, United Kingdom

## Abstract

Understanding emotion is critical for a science of healthy and disordered brain function, but the neurophysiological basis of emotional experience is still poorly understood. We analyzed human brain activity patterns from 148 studies of emotion categories (2159 total participants) using a novel hierarchical Bayesian model. The model allowed us to classify which of five categories—fear, anger, disgust, sadness, or happiness—is engaged by a study with 66% accuracy (43-86% across categories). Analyses of the activity patterns encoded in the model revealed that each emotion category is associated with unique, prototypical patterns of activity across multiple brain systems including the cortex, thalamus, amygdala, and other structures. The results indicate that emotion categories are not contained within any one region or system, but are represented as configurations across multiple brain networks. The model provides a precise summary of the prototypical patterns for each emotion category, and demonstrates that a sufficient characterization of emotion categories relies on (a) differential patterns of involvement in neocortical systems that differ between humans and other species, and (b) distinctive patterns of cortical-subcortical interactions. Thus, these findings are incompatible with several contemporary theories of emotion, including those that emphasize emotion-dedicated brain systems and those that propose emotion is localized primarily in subcortical activity. They are consistent with componential and constructionist views, which propose that emotions are differentiated by a combination of perceptual, mnemonic, prospective, and motivational elements. Such brain-based models of emotion provide a foundation for new translational and clinical approaches.

## Author Summary

Neuroimaging provides a unique way of understanding the ‘emotional brain’ by identifying patterns across multiple systems that imbue each instance of emotion with its particular qualities. In this meta-analysis across 148 studies, we ask whether it is possible to identify patterns that differentiate five emotion categories—fear, anger, disgust, sadness, and happiness—in a way that is consistent across studies. Our analyses support this capability, paving the way for brain markers for emotion that can be applied prospectively in new studies and individuals. In addition, we investigate the anatomical nature of the patterns that are diagnostic of emotion categories, and find that they are distributed across many brain systems associated with diverse cognitive, perceptual, and motor functions. For example, among other systems, information diagnostic of emotion category was found in both large, multi-functional cortical networks and in the thalamus, a small region composed of functionally dedicated sub-nuclei. Thus, rather than relying on measures in single regions, capturing the distinctive qualities of different types of emotional responses will require integration of measures across multiple brain systems. Beyond this broad conclusion, our results provide a foundation for specifying the precise mix of activity across systems that differentiates one emotion category from another.

## Introduction

Emotions are at the center of human life. Emotions play a crucial role in forging and maintaining social relationships, which is a major adaptation of our species. They are also central in the diagnosis and treatment of virtually every mental disorder [1]. The autonomic and neuroendocrine changes that accompany emotional episodes may also play an important role in physical health via peripheral gene expression and other pathways (e.g., [2]).

Because of their broad relevance, developing models of brain function to characterize and predict emotional experience is of paramount importance in the study of health and behavior. In animals, substantial progress has been made in linking motivated behaviors such as freezing (e.g. [3]), flight (e.g. [4]), reward pursuit (e.g. [5]), and aggressive behavior (e.g. [6]) to specific brain circuits. However, emotional experiences in humans are substantially more complex. Emotions such as fear emerge in response to complex situations that include basic sensory elements such as threat cues [7] as well as mental attributions about context information (e.g., the belief that one is being socially evaluated [8] and one’s own internal states [9,10]. A specific emotion category, like fear, can involve a range of behaviors, including freezing, flight, aggression, as well as complex social interactions. Thus, in spite of groundbreaking advances in understanding the circuitry underlying basic behavioral adaptations for safety and reproduction (including ‘threat’ behaviors; [11]), there is no comprehensive model of the neurophysiological basis of emotional experience in humans.

While at first blush it might seem that we know a lot about the brain processes underlying specific types of emotional experience, such as ‘anger,’ ‘sadness,’ ‘happiness,’ etc., it is not obvious that any brain pattern *specific* to an emotion category and *reproducible across studies* exists. The first two decades of neuroimaging saw hundreds of studies of the brain correlates of human emotion, but a central problem for the field is that the regions most reliably activated—e.g., the anterior cingulate, anterior insula, amygdala, and orbitofrontal cortex—are activated in multiple categories of emotions [12], and during many other sensory, perceptual and cognitive events [11,13]. Thus, activation of these regions is not specific to one emotion category or even emotion more generally. And, while there are many findings that seem to differentiate one emotion type from another, it is not clear that these findings are reliable enough (with sufficiently large effects) or generalizable enough across studies to meaningfully use brain information to infer what type of emotion was experienced.

Recently, studies have begun to take a pattern-based view, using multivariate pattern analyses to ‘decode’ affective and emotional experiences [14–18] and related affective psychopathology [19–21]. For example, in an innovative recent study, Kassam and colleagues [14] identified patterns of fMRI activity that distinguished multiple emotion categories. Though very promising, such approaches are limited in two basic ways. First, they are not really models of the generative processes sufficient to characterize a particular type of emotional experience. For example, the most common method uses Support Vector Machines to discriminate affective conditions (e.g., depressed patients vs. controls; [19]), and to discriminate 5 emotions, 10 separate maps (5 choose 2) are required for ‘brute-force’ pattern separation. Other models, such as the Gaussian Naïve Bayes approach [14], rely on differnces in activity patterns without capturing any of the interactions among brain regions that are likely critical for differentiating affective states [20,22,23]. Secondly, like univariate brain mapping, these approaches are beginning to yield a collection of patterns that seem to differentiate one affective state or patient group from another, but it remains to be seen how generalizable these predictive models are across studies, task variants, and populations. If history is a guide, in the area of emotion, the patterns that appear to reliably distinguish emotion categories may vary from study to study (e.g., [24] vs. [25]), making it difficult to identify generalizable models of specific emtion types.

The study presented here directly addresses both these issues. In this paper, our goal was to develop a generative, brain-based model of the five most common emotion categories—fear, anger, disgust, sadness, and happiness—based on findings across studies. Developing such a model would provide a rich characterization of the ‘core’ brain activation and co-activation patterns prototypical of each emotion category, which could be used to both make inferences about the distinctive features of emotion categories and their functional similarities across the brain or in specific systems. In addition, a useful model should be able to go beyond identifying significant differences across emotion categories and provide information that is actually diagnostic of the category based on observed patterns of brain activity. From a meta-analytic database of nearly 400 neuroimaging studies (6,827 participants) on affect and emotion, we used a subset of studies (148 studies) focused on the five emotion categories mentioned above to develop an integrated, hierarchical Bayesian model of the functional brain patterns underlying them.

We used this model to address two broad questions that have been of sustained interest in emotion research, and which are fundamental to the development of a more complete model of emotional experience. First, we asked whether it is possible to identify patterns of brain activity diagnostic of emotion categories across contexts and studies. Second, we asked whether emotion categories can be localized to specific brain structures or circuits, or to more broadly distributed patterns of activity across multiple systems. For many decades, scientists have searched to no avail for the brain basis of emotion categories in specific anatomical regions—e.g., fear in the amygdala, disgust in the insula, etc. The amygdala and insula are *involved* in fear and disgust, but are neither sufficient nor necessary for their experience. Conversely, emotions in both categories engage a much wider array of systems assumed to have cognitive, perceptual, and sensory functions [12], and damage to these systems can profoundly affect emotionality [26,27]. This multi-system view of emotion is consistent with network-based theories of the brain’s functional architecture [28,29] that have gained substantial traction in recent years. Based on these findings, we predicted that anger, sadness, fear, disgust and happiness emerge from the interactions across distributed brain networks that are not specific to emotion *per se*, but that subserve other basic processes, including attention, memory, action, and perception, as well as autonomic, endocrine, and metabolic regulation of the body [30,31]. Empirical support for this network approach to emotion has begun to emerge in individual experiments (e.g., [14,32–36]), and in task-independent (“resting-state”) analyses [37]. In Kassam et al. [14], for example, emotion category-related fMRI activity was widely distributed across the brain; and the same is true for recent work predicting depression from brain activity [19], again illustrating the need for a network approach.

Meta-analysis is uniquely suited to addressing our two questions because it examines findings from many studies and laboratories that utilize different procedures, stimuli, and samples. Our analysis included 148 PET and fMRI studies published from 1993 to 2011 (377 maps, 2159 participants) that attempted to specifically cultivate one of the five most commonly studied categories of emotion—happiness, fear, sadness, anger, and disgust. The studies were relatively heterogeneous in their methods for eliciting emotion (the most common were visual, auditory, imagery, and memory recall), and in the stimuli used (faces, pictures, films, words, and others). There was some covariance between emotion categories and elicitation methods (S1 Table), and we assessed the impact of this in several analyses. Studies used both male and female participants, primarily of European descent. The goal of our analysis was to test whether each emotion category has a unique signature of activity across the brain that is consistent despite varying methodological conditions (ruling out the possibility that emotion activity maps differ systematically because of method variables), providing a provisional brain ‘signature’ for each emotion category.

### The Bayesian Spatial Point Process (BSPP) Model

To develop a model for emotion categories and test its accuracy in diagnosing the emotions being cultivated in specific studies, we constructed a generative, Bayesian Spatial Point Process (BSPP) model of the joint posterior distribution of peak activation locations over the brain for each emotion category (see Methods and [38]). The BSPP model is a hierarchical Bayesian representation of the joint density of the number and locations of peak activations within a study (i.e., x, y, z coordinates) given its particular emotion category. The BSPP model differs from standard univariate [12,13,39] and co-activation based [40,41] approaches to meta-analysis in several fundamental ways. For instance, Activation Likelihood Estimation (ALE), multi-level kernel density analysis (MKDA), and co-activation approaches are 1) not generative models of the emotion, and 2) not multivariate in brain space. Because they are not generative models, standard analyses provide only descriptive, summary maps of activity or bivariate co-activation for different psychological states.

The BSPP, by contrast, can be used to predict the number and locations of activation in a new study given its emotion category and the probability that a new study will contain peak activations within a particular region or regions. The generative (or ‘forward’) model estimates a set of brain locations, or ‘population centers’, that are consistently active during instances of a given emotion category. Stochastic sampling from these population centers with study-level variation (in methods, pre-processing, statistical analysis, etc.) and measurement-level spatial noise is assumed to generate the observed data. The result is a rich, probabilistic representation of the spatial patterns of brain activity associated with each emotion category. Once estimated, the model can be used to [1] investigate the brain representations for each emotion implicit in the model and [2] infer the most likely emotion category for a new study based on its pattern of activation (‘reverse’ inference).

The generative model concerns the process by which emotional instances of a given category produce observed peak activation foci, and the likelihood with which they do so. Activation data from studies or individuals are modeled at three hierarchical levels (see Methods and [38] for more details). At Level 1 is the individual study data, in the form of peak coordinate locations. Level 2 models the activation centers across study with a data-generating focus that can result in variable numbers of reported locations depending on the smoothness in the image and analysis/reporting choices. Level 3 models the location of ‘true’ population centers for each emotion category with a probability distribution over space specified by the model.

**Fig 1:**
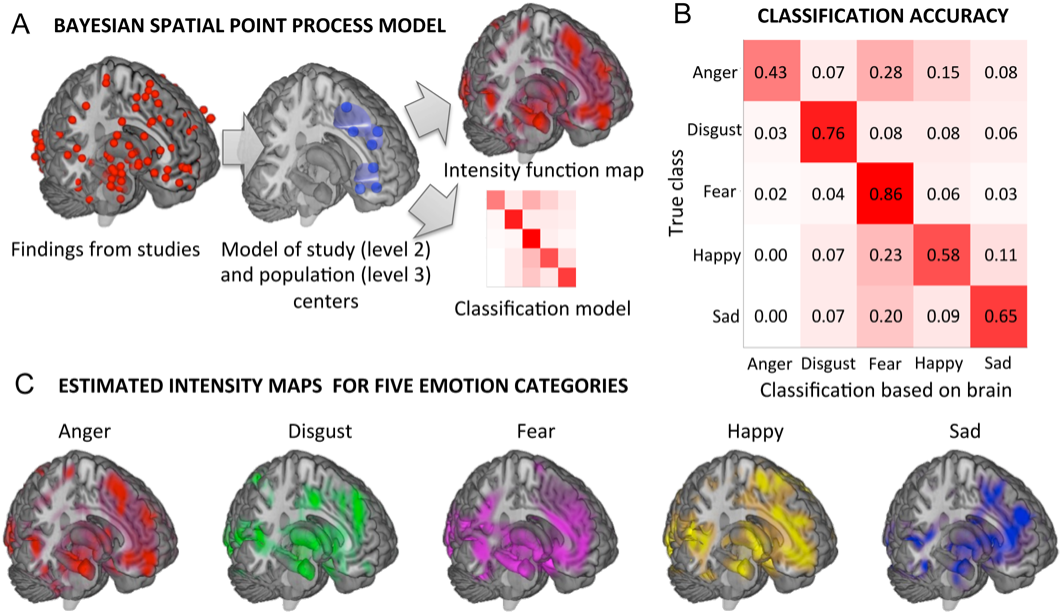
Classification of emotion category using the Bayesian Spatial Point Process model. A) A schematic of the method, which models the population density of activation across the brain with a sparse set of multivariate Gaussian distributions at two levels (study center and population center). The intensity function map summarizes the expected frequency of activation conditional on an emotion category. The model also represents the joint activation across multiple brain regions, which is not captured in the intensity map. The model can also be used for classification by calculating the conditional likelihood of each emotion category given a set of foci using Bayes’ rule. B) Confusion matrix for the 5-way classification of emotion category based on the model. Diagonal entries reflect classification accuracy. C) The intensity maps for each of the 5 emotion categories. Intensity maps are continuous over space, and their integral over any portion of the brain reflects the expected number of activation centers in that area for all studies with a particular emotion. The maps are thresholded for display at a voxel-wise intensity of 0.001 or above.

The model parameters—including the number and locations of population centers and spatial variation at study and peak levels—were estimated by fitting the model to peak activation coordinates from our database using Markov Chain Monte Carlo (MCMC) sampling with a generative birth-and-death algorithm for population centers. The MCMC procedure draws samples from the joint posterior distribution of the number and locations of peak activations in the brain given an emotion category. The posterior distribution is summarized in part by the *intensity function map* representing the spatial posterior expected number of activation or population centers in each area across the brain given the emotion category; this can be used to interpret the activation pattern characteristic of an emotion category (Fig. 1A). Since the BSPP models the *joint* distribution of the number and locations of a *set* of peak coordinates, the posterior distribution also includes information about the co-activation across voxels; thus, MCMC samples drawn from it can be used to infer on the co-activation patterns and network properties for each emotion category (discussed below).

## Results

### Predicting Emotion Categories from Patterns of Brain Activity

We applied the BSPP model to ‘decode’ each study’s emotion category from patterns of brain activity in our meta-analytic database. Once the Bayesian model is estimated, it can be inverted in a straightforward manner to estimate the posterior probability of each emotion category given a set of brain activation coordinates (see Methods). We used Bayes rule to obtain these probabilities, assuming no prior knowledge of the base-rate of studies in each category (i.e., flat priors), and used leave-one-study-out cross-validation so that predictions were always made about studies not used to train the model. The model performed the five-way classification of emotion categories with accuracy ranging from 43% for anger to 86% for fear to (mean balanced accuracy = 66%; Fig. 1B; S1 Table); chance was 20% for all categories, and absence of bias was validated by a permutation test. The BSPP model outperformed both a Naïve Bayes classifier (mean accuracy was 35%) and a nonlinear support-vector-machine based classifier (mean accuracy was 33%; see Supplementary Methods for details), confirming its utility in distinguishing different emotion categories.

Next, we examined whether the covariance between emotion categories and the methods and stimuli used to induce emotion (S1 Fig) contaminated the classification accuracies. For instance, 23% of the studies used recall and 50% used visual images to induce sadness, whereas 2% of studies used recall and 90% used visual images to induce fear. Thus, patterns for sadness vs. fear might be differentiable because the different stimuli elicit different brain responses. We verified that classification results were essentially unaffected by controlling for induction method (40–83% accuracy across the five emotion categories, and 61% on average; S1 Table). We also attempted to predict the emotion category using several methodological variables, including the method of elicitation (the most common were visual, auditory, imagery, and memory recall), stimulus type (faces, pictures, films, words, and others), participant gender, control condition, and imaging technique (PET or fMRI). Several of these variables accurately classified some emotion categories in the five-way classification (S2 Table), but no methods variable performed as well as the original BSPP model in accuracy. Stimulus type, task type, and imaging technique predicted emotion significantly above chance, at 32%, 26%, and 26% accuracy, respectively. Elicitation method, participant sex, and control condition for the emotion contrasts were at 24%, 21%, and 18%, respectively, all non-significant). Thus, although there are some dependencies between the methods used and the emotion categories studied, the emotion category patterns that we identified with our BSPP approach appeared to generalize across the different methods (at least as represented in our sample of studies).

Fig. 1C shows the intensity maps associated with each emotion category. The distinctiveness for each emotion category was distributed across all major regions of the cortex, as well as in subcortical regions, as supported by additional analyses described below. Notably, limbic and paralimbic regions such as the amgydala, ventral striatum, orbitofrontal cortex (OFC), anterior cingulate cortex (ACC), brainstem, and insula were likely to be active in *all* emotion categories, though with different and meaningful distributions within each region (as shown in analyses below). In addition, regions typically labeled as ‘cognitive’ or ‘perceptual’ were also engaged and potentially differentially engaged across categories, including ventromedial, dorsomedial, and ventrolateral prefrontal cortices (vmPFC, dmPFC, and vlPFC), posterior cingulate (PCC), hippocampus, and medial temporal lobes, and occipital regions.

### Emotion-Predictive Brain Patterns: Relationships to Known Intrinsic Networks

**Fig 2:**
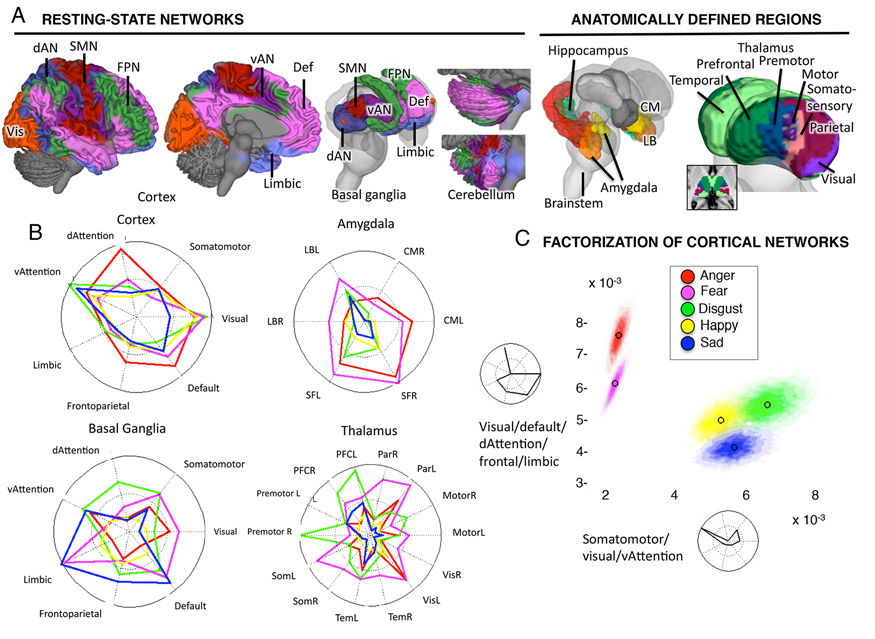
Emotion-predictive patterns of activity across cortical networks and subcortical regions. A) Left: Seven resting-state connectivity networks from the Buckner Lab with cortical, basal ganglia, and cerebellar components. Colors reflect the network membership. Right: Published anatomical parcellations were used to supplement the resting-state networks to identify sub-regions in amygdala (131), hippocampus (131, 132), and thalamus (133). dAN: dorsal attention network; Def: default mode network; FPN: fronto-parietal network; Limbic: limbic network; SMN: somatomotor network; vAN: ventral attention network; Vis: visual network. B) The profile of activation intensity across the 7 cortical and basal ganglia resting-state networks, and anatomical amygdalar and thalamic regions. Colors indicate different emotion categories, as in Fig. 1. Red: anger; green: disgust; purple: fear; yellow: happiness; blue: sadness. Values farther toward the solid circle indicate greater average intensity in the network (i.e., more expected study centers). C) Two canonical patterns estimated using non-negative matrix factorization, and the distribution of intensity values for each emotion across the two canonical patterns. The colored area shows the 95% joint confidence interval (confidence ellipsoids) derived from the 10,000 Markov chain Monte Carlo samples in the Bayesian model. Non-overlapping confidence ellipsoids indicate significant differences across categories in the expression of each profile.

To characterize the BSPP intensity maps, we calculated the mean intensity for each emotion category in 49 a priori regions and networks, which together covered the entire brain (Fig. 2A). For cortical, basal ganglia, and cerebellar networks, we used results from Buckner and colleagues [42–44], who identified seven networks with coherent resting-state connectivity across 1,000 participants. Each of the seven included a cortical network and correlated areas within the basal ganglia (BG) and cerebellum. We supplemented these networks with anatomical sub-regions within the amygdala, hippocampus, thalamus, and the brainstem and hypothalamus. We tested whether a) broad anatomical divisions (e.g., cortex, amygdala) showed different overall intensity values across the five emotion categories; and b) whether the ‘signature’ of activity across networks within each division differed significantly across emotions (S3 Table). Our broad goal, however, was not to exhaustively test all emotion differences in all regions, but to provide a broad characterization of each emotion category and which brain divisions are important in diagnosing them.

#### Cortical patterns

Overall, different emotion categories involved reliably different patterns of activation across these anatomically circumscribed zones (Figs. 2B, S3 and S3 Table), and illustrate how the BSPP model can be used to draw inferences across multiple anatomical levels of analysis.

There are three salient features of the overall cortical architecture for emotion categories. First, there were few differences among emotion categories in the overall level of cortical engagement (summarized in S3 Table). Second, no emotion category mapped to any single cortical network, but emotion categories could be distinguished by significant differences in their profiles across networks (Fig. 2B, p <. 001 overall; S3 Table). To identify patterns across the seven cortical networks, we used non-negative matrix factorization [45] to decompose these intensity values into two distinct profiles (Fig. 2C). These profiles were differentially expressed by different emotions (p < 0.01) and showed significant differences in 8 of the 10 pairwise comparisons across emotions (q < 0.05 FDR corrected; S3 Table). Adopting Buckner et al.’s terminology for the networks, we found that anger and fear categories were characterized by a profile that included mainly involved the ‘dorsal attention,’ ‘visual’ (occipital), ‘frontoparietal,’ ‘limbic,’ and ‘default mode’ networks. Patterns for disgust, sadness, and happiness categories were characterized by moderate activation of this profile and high intensity in a second profile that included the ‘ventral attention,’ ‘somatomotor,’ and ‘visual’ networks (Fig. 2C). When combined, these two profiles differentiated all five emotion categories to some degree, as can be seen in the nearly non-overlapping probability density functions (colored regions in Fig. 2C). Third, the grouping of emotion categories in terms of cortical activity profiles did not match folk conceptions of emotions (e.g., [46]) or the dimensions identified in behavioral emotion research [47]. For example, happiness and disgust categories (one ‘positive’ and one ‘negative’ emotion) produced very similar profiles, but disgust and fear categories (both high-arousal negative emotions) produced very different profiles.

#### Subcortical patterns

Different patterns across emotion categories were also discernable in subcortical zones including the amygdala, thalamus, brainstem/cerebellum, and basal ganglia (Fig. 2B). Non-negative matrix factorization again produced distinct profiles for each emotion category (S3 Fig), reveals several additional characteristics over and above the cortical profiles. First, as with cortical networks, the largest differences were not overall intensity differences across emotion categories, but rather the profile of differences across sub-regions. Even the zones most differentially engaged in terms of average intensity, such as the amgydala (Fig. 2B), showed appreciable intensity in all five emotion categories (see also Fig. 1), consistent with previous meta-analyses. Second, whereas cortical networks discriminated the fear and anger categories from the other emotions, hippocampal and cerebellar/brainstem profiles discriminated fear from anger (q < 0.05 FDR; S3 Fig and S3 Table). Third, the relationships between cortical, BG, and cerebellar networks varied across emotion categories. For example, the anger category produced the highest intensity in the cortical ‘dorsal attention’ network, whereas in the BG ‘dorsal attention’ zone, the disgust category was high and the anger category was low (Fig. 2B). This suggests that the network coupling as observed in task-independent data (i.e., ‘resting-state’) is not preserved when emotional experiences are induced. Thalamic areas connected with motor and premotor cortices were most activated in fear and disgust categories, as were ‘somatomotor’ BG regions, but the ‘somatomotor’ cortical network is low in disgust and fear categories. These patterns suggest that simple characterizations such as more vs. less motor activity are insufficient to characterize the brain representations of emotion categories.

## Network Co-activation Differences among Emotion Categories

One of the important differences between the BSPP model and previous meta-analytic approaches is that is it sensitive to co-activation patterns across regions. By saving the average intensity values for each region/network from each MCMC iteration, we were able to estimate the co-activation intensity as the correlation between average intensity values for each pair of regions. We used a permutation test to threshold the co-activation estimates (using the most stringent of the q <. 05 FDR-corrected thresholds across categories).

**Fig 3:**
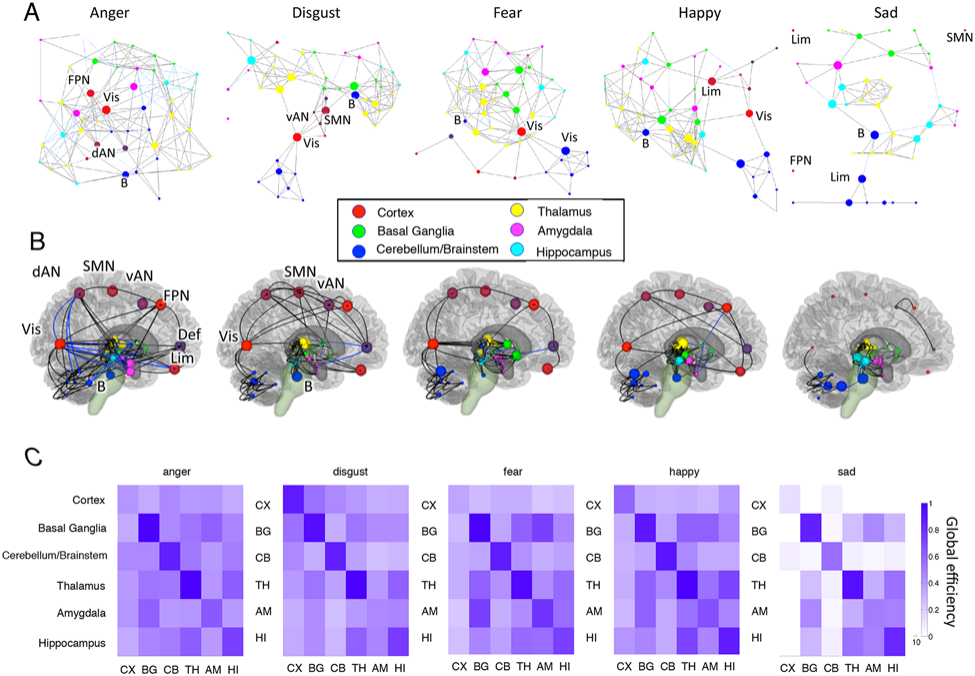
Co-activation graphs for each emotion category. A) Force-directed graphs for each emotion category, based on the Fruchterman-Reingold spring algorithm (134). The nodes (circles) are regions or networks, color-coded by anatomical system. The edges (lines) reflect co-activation between pairs of regions or networks, assessed based on the joint distribution of activation intensity in the Bayesian model (Pearson’s r across all MCMC iterations) and thresholded at P <. 05 corrected based on a permutation test. The size of each circle reflects its betweenness-centrality (48, 49), a measure of how strongly it connects disparate networks. (B) The same connections in the anatomical space of the brain. One location is depicted for each cortical network for visualization purposes, though the networks were distributed across regions (see Fig 3A). C) Global network efficiency (see refs. (135, 136)) within (diagonal elements) and between (off-diagonals) brain systems. Global efficiency (135, 136) is defined as the inverse of the average minimum path length between all members of each group of regions/nodes. Minimum path length is the minimum number of intervening nodes that must be traversed to reach one node from another, counting only paths with statistically significant associations and with distance values proportional to (2—Pearson’s r), rather than binary values, to better reflect the actual co-activation values. Higher efficiency reflects more direct relationships among the systems. Values of 0 indicates disjoint systems, with no significant co-activation paths connecting any pair of regions/networks, and values of 1 indicate the upper bound of efficiency, with a perfect association between each pair of regions. Co-activation is related to connectivity and network integration, though all fMRI-based connectivity measures only indirectly reflect actual neural connections. Efficiency is related to the average correlation among regions (r = 0.76) but not the average intensity (r = 0.02; see S5 Fig).

Fig. 3 shows that each emotion category was associated with a qualitatively different configuration of co-activation between cortical networks and subcortical brain regions. In Fig. 3A, force-directed graphs of the relationships among the 49 anatomical regions/networks demonstrate very different topological configurations for the five emotion categories. In these graphs, regions and networks are represented by circles (nodes), with significant co-activations (edges) represented as lines. The size of each circle reflects the region/network’s *betweeness centrality* [48,49], a graph-theoretic measure of the degree to which a region/network is a ‘connector’ of multiple other regions. Colors reflect membership in six cerebral zones: Cortex, basal ganglia, cerebellum/brainstem, thalamus, amygdala, and hippocampus. Fig. 3B shows the same graph’s relationships in anatomical brain space. Finally, Fig. 3C shows estimates of average co-activation within (diagonals) and between (off-diagonals) the six cerebral zones. The co-activation metric reflects global efficiency, based on the average shortest path length, a graph theoretic measure of the shortest path connecting the regions in the co-activation graphs, and was calculated as the average (1/path length) between pairs of regions/networks.

Emotion patterns were distinguished by both their patterns of co-activation and by the regions that are ‘connectors.’ The *anger* category is characterized by relatively strong and uniform connections across cerebral zones compared to other emotion categories, with strong co-activation among cortical, basal ganglia, and cerebellar networks, and other regions, particularly the right amygdala. Connectors (>90^th^ percentile in betweenness-centrality) include the cortical frontoparietal network, right amygdala, and brainstem. (Visual cortex was a connector for all emotion categories except sadness). In *disgust*, by contrast, cortical networks connect to basal ganglia regions and serve as a bridge to an otherwise isolated cerebellum. Connectors include the somatomotor basal ganglia network and brainstem. The *fear* category is marked by reduced co-activation among cortical networks and between cortex and other structures, but the basal ganglia are tightly integrated with the amygdala and thalamus. In *happiness*, intra-cortical co-activation is higher, but cortical-subcortical co-activation is low, and connectors include the limbic cortical network, motor thalamus, and visual basal ganglia and cerebellum. *Sadness* is characterized by dramatically reduced co-activation within the cortex, between cortex and other regions, and between cerebellum and other regions. Intra-thalamic, intra-basal ganglia, and intra-cerebellar co-activation are relatively preserved, but large-scale connections among systems are largely absent. Connectors include the limbic cerebellum, brainstem, two hippocampal regions, and the left centromedial amygdala.

## Discussion

The results of our BSPP model indicate that emotion categories are associated with distinct patterns of activity and co-activation distributed across the brain, such that there is a reliable brain basis for diagnosing instances of each emotion category across the variety of studies within our meta-analytic database. The brain patterns are sufficient to predict the emotion category targeted in a study with moderate to high accuracy, depending on the category, in spite of substantial heterogeneity in the paradigms, imaging methods, and subject populations used.

The accuracy levels for predicting emotion category (43–86%) are substantially above chance (20%) but below 100%, though they may be close to the limit that can be obtained using such a heterogeneous population of studies, particularly given power issues due to small sample sizes in most studies. In addition, the results have much greater specificity than the two-choice classifications that are most common in fMRI studies. For example, though anger has only a 43% sensitivity, it has 99% specificity. In addition, the positive predictive value is above 60% and negative predictive value above 89% for all categories (see Table 1). This means that if an emotion is classified as an instance of a particular category, there is at least a 60% chance that it truly belongs to the category; and if not classified as an instance of a category, there is at least a ~90% chance that it truly is not an instance.

**Table 1:** Population centers and 5-way emotion-classification performance.

	Anger	Disgust	Fear	Happy	Sad
**Number of population centers**					
Mean	51.53	35.68	40.4	31.47	34.51
SD	2.64	2.44	1.99	2.26	2.16
Median	52	36	40	31	35
UCL	46	31	36	27	30
LCL	57	41	44	36	39
**Model Performance**					
Sensitivity	.43	.76	.86	.59	.65
Specificity	.99	.94	.80	.91	.94
PPV	.89	.74	.63	.62	.67
NPV	.89	.95	.94	.90	.93

**Note**: Number of population centers refers to the estimated number of discrete brain regions activated for each emotion category. SD denotes standard deviation, and UCL and LCL denote upper and lower 95% posterior credible intervals, respectively. Model performance includes the hits rate (sensitivity), correct rejection rate (specificity), positive and negative predictive values (PPV and NPV) for a test classifying each study as belonging to an emotion category or not based on its reported brain foci. Performance statistics are based on leave-one-study-out cross-validated results.

However, the major value of the model is not merely in inferring the emotion category from brain data in new studies, but in characterizing a canonical, population-level representation of each emotion category that can constrain the development of theories of emotion and brain-based modeling and prediction in individual studies. Whereas emotion-predictive features developed by multivariate pattern analyses (MVPA) within individual studies can be driven by task- or subject-related ideosyncracies and fail to generalize, a strength of the representations we have identified is that, because they were trained across heterogeneous instances, they are likely to reflect generalizable features. Below, we discuss the value of the generative BSPP model as a computational technique for characterizing emotion, and the implications for theories of emotion and brain network science.

### The Value of the Generative BSPP Model as a Computational Approach

Because it is a generative model, the BSPP model of emotion categories is capable of making predictions about new instances. Other methods—such as our previous MKDA analyses, ALE analyses, and bivariate co-activation analyses that we and others have developed—are not generative models, and would not be expected to be appropriate to or perform well in classification.

In addition, unlike 'brute force' pattern classification algorithms, we can classify emotions with a single, generative representation of each emotion category. For example, when using Support Vector Machines to discriminate the five categories, ten separate classifier maps (5-choose-2) are required to predict the category, rather than relying on a single representation of each category and the likelihood that a particular study belongs to it. In addition, the nonlinear SVM model we adapted for meta-analytic classification performs substantially more poorly in classification.

In the BSPP model, each representation includes information about both activation and co-activation across systems. However, unlike data-driven pattern classification models, this model can be queried flexibly—i.e., here, we present graphs of bivariate (2-region) co-activation strengths, but other, more comprehensive summaries can be used, including those that were not explicitly used in model training. For example, we demonstrate this by using non-negative matrix factorization (NNMF) to derive canonical profiles of activation across cortical and subcortical systems. We then re-calculate the model likelihood according to the new metric of canonical profile activation, without re-fitting the model, and are able to make statistical inferences about the differences among emotion categories in that new feature space. This flexibility is a hallmark of generative Bayesian models that provides advantages in allowing researchers to test new metrics, features, and patterns, rather than being limited to a fixed set of features such as pair-wise correlations.

Beyond these considerations of methodology and broad interpretation, the present findings bear on theories of emotion, and the ways in which studies look for the hallmarks of particular emotional experiences, in novel and specific ways. We elaborate on some of these below.

### New Implications for Emotion Theories

#### External vs. internal response demand: A new way of grouping emotions

The present findings constitute a brain-based description of emotion categories that does not conform to emotion theories that are based on the phenomenology of emotion. Our findings do not support basic emotion theories [46], which are inspired by our phenomenology of distinct experiences of anger, sadness, fear, disgust and happiness that should be mirrored in distinct modules that cause each emotion. According to such theories, each emotion type arises from a dedicated population of neurons that are [1] architecturally separate, [2] homologous with other animals and [3] largely subcortical or paralimbic (e.g., infralimbic-amygdala-PAG). Many theories also assume that the signature for an emotion type should correspond to activation in a specific brain region or anatomically modular circuit (e.g., [50]), usually within subcortical tissue (e.g., [51]). In a recent review of basic emotion theories, Tracy wrote that the “agreed-upon gold standard is the presence of neurons dedicated to an emotion’s activation” ([52], p. 398).

Our findings do not support theories that adhere to those tenets. The areas of the brain sufficient to represent and classify emotion category in our results are not architecturally separate, and include cortical networks that may not have any direct homologue in nonhuman primates [53]. Our results suggest these cortical networks act as a bridge between subcortical systems in different ways, depending on the emotion category, which is consistent with the anatomy and neurophysiology of cortical-subcortical circuits (e.g., [54]). Though we do not have the resolution to examine small, isolated populations of cells (see below for more discussion), we are not aware of findings that identify single neurons dedicated to one specific type of emotion within prefrontal, somatosensory, and other networks. Thus, the weight of evidence suggests that cortical networks are centrally and differentially involved in emotion generation and that they are not conserved across species.

However, if our findings do not conform to predictions from basic emotion theories, nor do our findings support so-called ‘dimensional approaches’ to emotion based on phenomenological experience [47,55,56]. Valence, arousal, and approach-avoid orientation are descriptors that are fundamental at the phenomenological level, but not necessarily at the level of brain architecture that we studied here. Thus, theories of emotion have been underconstrained at the neurophysiological level, with an absence of specific human brain data on the necessary and sufficient conditions to differentiate across emotion categories, and our findings can inform the evolution of emotion theories in specific ways.

We found that in the cortex, anger and fear categories are very similar, and preferentially activate 'dorsal attention,' fronto-parietal,' and 'default mode' networks (as defined by resting-state connectivity). Happiness, sadness, and disgust categories belong to another, distinct group, with preferential activity in somatomotor and 'ventral attention' (or 'salience') networks. This distinction is pronounced and strong in this dataset, and it cannot be explained by the methodological variations across studies that we examined. Importantly for emotion theories, neither can it be explained by traditional emotion concepts: The 'ventral attention' group includes two negative emotions (disgust and sadness) and one positive one (happiness), and one most often labeled as high-arousal (disgust) and two as low-arousal (happiness and sadness), at least with respect to the in-scanner paradigms typically used in these studies (that is, sadness can be high-arousal, but in-scanner sadness manipulations are typically low-arousal). One emotion is traditionally categorized as approach-related (happiness), and two as avoidance-related (sadness and disgust). The 'dorsal attention' group contains two negative emotions, one traditionally categorized as approach (anger) and one as avoidance-related (fear). Thus, the structure that emerges by examining the cortical patterns of activity across emotion categories is not likely to be explainable in terms of any of the traditional phenomenological dimensions used in emotion theories.

This pattern of findings suggests a potential new dimension around which emotional brain systems may be organized. The 'dorsal attention' and 'fronto-parietal' networks are consistently engaged by tasks that require the allocation of attentional resources to the external world, particularly as guided by task goals [57,58]. By contrast, the ventral attention network (which is largely spatially overlapping with the so-called salience network [59]) includes (a) more ventral frontoparietal regions consistently engaged during exogenously cued, more automatic processing of events, and (b) cingulate, insular, and somatosensory regions (e.g., SII) that are targets of interoceptive pathways that carry information about pain, itch, and other visceral sensations (for reviews, see [30,60]). The default mode network may provide a bridge between conceptual cortical processes and visceromotor, homostatic, and neuroendocrine processes commonly associated with affect [61], including the shaping of learning and affective responses based on expectations [62]. It is consistently engaged during conceptual processes such as semantic memory [63], person perception [64,65], and prospection about future events [66], as well as in emotion [36,67], valuation, and conceptually driven autonomic and neuroendocrine responses [68–70] and their effects on cognition [71]. Thus, the modal patterns we observed suggest that anger and fear categories preferentially engage cortical processes that support an 'external orientation/object focused' schema, characterized by goal driven responses where objects and events in the world are in the foreground. By contrast, sadness, happiness, and disgust engage cortical patterns that support an internal orientation/homeostatic focused schema, characterized by orientation to immediate somatic or visceral experience, which prioritizes processing of interoceptive and homeostatic events.

In sum, the new dimension of goal driven/external object focused vs. reactive/internal homeostasis-focused, rather than traditional phenomenological dimensions, may be important for capturing distinctions between emotion categories respected by gross anatomical brain organization.

#### Further implications for dimensional models: Re-thinking the nature of valence

The importance of the external/object versus internal/interoceptive dimension is also reflected in the surprising observation that our attempts to classify positive versus negative valence across the entire set largely failed. The finding that emotion categories are a better descriptor than valence categories provides new information about how emotion categories are represented in brain systems. We are in no way claiming that positive and negative valence is unimportant. At the microscopic level, separate populations of neurons within the same gross anatomical structures appear to preferentially encode positively versus negatively valenced events [72–74]. Valence may be an aspect of emotional responses that is particularly important subjectively, but is not the principal determinant of which brain regions are engaged during emotional experience at an architectural level. By analogy, the loudness of a sound has important subjective and behavioral consequences; but the brain does not contain a separate "loud sound system" and "soft sound system." Because valence is important, it has been widely assumed that the brain must contain separate systems for positive and negative valence. Our results suggest they may be aspects of processing within emotion systems. In support of this view, recent work has demonstrated that emotions frequently thought of as univalent, such as sadness, can be experienced as either positive or negative, depending on the context.

#### The importance of specific, differentiated patterns of cortical-subcortical interactions

Though we focus mainly on the cortex in our interpretations above, we are not claiming that cortical patterns alone are sufficient to fully characterize differences across emotion categories. Cortical-subcortical interactions have been central to emotion since the term ‘limbic system’ was coined (e.g., [75]). Subcortical responses are likely equally or more important, and show different organizational patterns. The pattern of cortico-cerebellar connectivity differs markedly across emotion categories (see Table 2): In anger, fronto-parietal cortex is co-activated positively with amygdala and cerebellum, and the dorsal attention network is negatively associated with cerebellar activation; in disgust, somatomotor cortex associations with basal ganglia dominate; in fear, visual-subcortical (esp. amygdala) co-activation dominates. And, perhaps most prominently, sadness is characterized by a profound lack of co-activation between cortical and subcortical cerebellar/brainstem networks, and a strong, preserved co-activation of hindbrain (cerebellar/brainstem) systems.

**Table 2:** Summary of brain features characterizing each emotion category.

Speculative Interpretation, psychological predictions/ inferences	Similarity	Coactivation patterns	Activity patterns	
Strong goal-driven attention component, with central cerebellar involvement for strong sensorimotor integration; lower ‘impulsive’ general motor priming than often assumed; anger studied in scanner is more calculated than impulsive	Cortical, amygdala pattern similar to fear; hippocampal and cerebellar pattern unique	Strong visual-to-frontoparietal cortex; strong cortico-cerebellar and cortico-amygdalar, mainly fronto-parietal and dorsal attention networks; strong subcortical coactivation	Strong dorsal attention, fronto-parietal cortico-cerebellar circuit; default-mode cortical activity; Relatively little basal ganglia	**Anger**
Strong cortical involvement, emphasizing ventral attention and somatosensory networks implicated in exogenously driven attention; Strong cortico-striatal coactivation may prioritize immediate action generation; low cerebellar involvement suggests less fine-grained control of responses	Cortical pattern similar to happiness and sadness, but stronger engagement; subcortical pattern in basal ganglia relatively unique	Strong somatomotor cortex to basal ganglia; low cerebellar and strong intracortical coactivation; visual-to-frontal cortex network coactivation is critical bridge integrating subcortical systems	Ventral attention network in cortex; dorsal attention in basal ganglia	**Disgust**
Fear as studied in scanner has strong visual-to-subcortical component; reduced demand on cortically driven planned responses/goal. Amygdala activity/co-activation strong, but dominated by basalateral complex implicated in cue-threat associative learning.	Cortical, amygdala pattern similar to anger; distinctive, bilateral hippocampal pattern	Weak cortical-subcortical coactivation except visual cortex, and weak intracortical coactivation, strong basal ganglia coactivation with amygdala and thalamus	Strong amygdala (basolateral) hippocampus; parietal and somatosensory thalamus; visual, default-mode, and limbic basal ganglia	**Fear**
Relatively low demand for integrated planning/action systems (somatosensory/cerebellar); Particularly strong limbic network implicated in psotive value and endogenously driven expectancies; Low amygdala involvement consistent with reduced reliance on exogenous cues.	Cortical pattern similar to sadness and disgust; distinctive left-sided hippocampal pattern	Strong within-system coactivation (cortex, basal ganglia, thalamus, cerebellum), but relatively weak cortical-subcortical coactivation	Low amygdala, thalamus, and basal ganglia activity; Left-sided hippocampus and medial temporal	**Happy**
Very weak activation of integrated planning/action systems (dorsal attention/cerebellar), and systems driven by exogenous cues (visual, amygdala); very weak cerebellar integration and system integration overall; reflexive cerebellar-brainstem responses strong and operate without co-activation with cortex	Cortical patterns similar to happiness and disgust; pattern in cerebellum and brainstem more similar to fear	Very weak intra-cortical and cortical-subcortical coactivation relatively isolated systems; strong cerebellar-brainstem coactivation, but weak cerebellar coactivation with other systems	Low amygdala, hippocampal, thalamic activity; Limbic, frontoparietal, and default basal ganglia networks; Limbic cerebellum	**Sad**

With regard to sadness, this pattern might suggest reduced higher brain control over evolutionarily ancient hindbrain visceromotor functions—which are otherwise capable of mediating many types of affective responses and even affective learning without the rest of the brain [76–78], producing a loss in flexibility. This pattern might provide hints as to why psychopathology, and depression in particular, frequently impairments in the ability to describe emotional experience in a fine-grained, contextualized manner (e.g., alexithymia), which is a risk factor for multiple psychiatric conditions (e.g., [79–83]). They also provide a new way of thinking about the reasons for the observed benefits of subgenual cingulate cortical stimulation for depression [84], as the subgenual cingulate and surrounding ventromedial prefrontal cortex have the densest projections to the brainstem of any cortical region [85,86].

Our findings on prefrontal-cerebellar-brainstem co-activation also illustrate how the present network-based study can provide new, theoretically and practically relevant information. In spite of the existence of topographically mapped prefrontal-cerebellar circuits [87] that play a prominent role in emotion as revealed by human brain stimulation and lesions (e.g., [88]), the prefrontal-cerebellar-brainstem axis has not been a major focus of recent theoretical and MVPA-based studies of depression (e.g., [17,89]) or emotion more generally (e.g., [14,15]), and is often specifically excluded from analysis. However, cerebellar connectivity plays a central role in some of the most discriminative whole-brain studies of depressed patients vs. controls to date [19,21]. However, these latter studies omitted the brainstem (and note that “the functional connectivity of the brainstem should be investigated in the future”). In our results, the brainstem is also critical: In sadness, it is co-activated with the cerebellum only, whereas in other emotions it is much more integrated with the thalamus, basal ganglia, and cortex. Thus, our results can help inform and guide future studies on this system in specific ways.

#### Towards a multiple-system view of emotion

Our findings place an important constraint on emotion theories that identify emotions with discrete brain regions or circuits. Since the inception of the 'limbic system' concept by Paul MacLean [75], it has been widely assumed that there is an 'emotional' brain system that encodes experience and is dissociable from systems for memory, perception, attention, etc. Our results provide a compelling and specific refutation of that view. Single regions are not sufficient for characterizing emotions: Amygdala responsivity is not sufficient for characterizing fear; the insula in not sufficient for characterizing disgust; and the subgenual cingulate is not sufficient for characterizing sadness. While other meta-analyses have reached this broad conclusion for individual brain regions, we also found that no single network (at least as currently defined from resting-state connectivity; such networks are widely used in inference and classification) is sufficient for characterizing an emotion category, either. Rather, the activity patterns sufficient to characterize an emotion category spanned multiple cortical and subcortical systems associated with perception memory, homeostasis and visceromotor control, interoception, etc.

The examples above provide specific illustrations of how particular interaction patterns are important for particular emotions. But, even beyond the examples we discuss, the rich patterns that emerge from our model can inform future studies of other emotion-specific interaction patterns. For example: The amygdala has been often discussed in relation to fear, but our results demonstrate that the preference for fear is limited mainly to the basolateral amygdalar complex, and other sub-regions closer to the basal forebrain may play consistent roles across multiple emotions. Happiness is characterized by low cortical co-activation with amygdala, thalamus, and basal ganglia, tight basal ganglia-thalamic integration, and a novel left-hemisphere dominance in the hippocampus that may be related to theories of lateralized valence processing [90,91] (Table 2). In some cases, the results are counterintuitive based on previous emotion theories, and in other cases they are consistent. For example, anger is widely thought to involve increased action tendencies, and based on this one might predict increased activation in somatomotor networks [92]. However, our results paint a picture more consistent with prefrontally mediated, goal directed regulation of motor systems. Conversely, sadness is associated with reduced feelings of somatomotor activity in the limbs [93], consistent with overall ‘de-energization’ and internal focus. Consistent with this, both major motor-control systems (basal ganglia and cerebellum) are more isolated from the cortex and limbic system in sadness than any other emotion.

More generally, the novel finding that each emotion category can be described at the population level by a pattern across multiple intrinsic brain networks provides a template for what the relevant interactions are for future studies. The broad architecture of these networks was predicted *a priori* by the Conceptual Act Theory [94], part of a new family of construction theories hypothesizing that anger, sadness, fear, disgust, and happiness are not biological types arising from dedicated brain modules, but arise from interactions of anatomically distrtibuted, core systems [30,31,94–96]; however, the specific patterns and inter-relationships involved are just beginning to be discovered. Even the broad principles of this architecture do not conform to predictions of basic emotion theories, nor of appraisal theories, which imply that there is one brain system corresponding to specific aspects of cognitive appraisal (e.g., valence, novelty, control, etc.). We believe that previous theories on emotion have been underconstrained by brain data, and the present findings constitute a specific set of constraints that may be integrated into future theories on emotion. In addition, the multi-network emotion representations we identify here paint a picture of emotion that underscores the importance of NIMH’s recent RDoCs approach, as well as recent papers taking a network approach to psychopathology [19,97–100]—and they provide a specific template for testing specific network-topological predictions about the ingredients of emotion and the category-level responses that emerge from their interactions.

### Implications of Accuracy Differences across Categories

In this study, the five way decoding accuracy for emotion categories varies substantially across categories. Fear was the most accurate overall, with 86% accuracy, whereas anger was the least accurate, at 43%. These findings could indicate heterogeneity in the categories themselves. However, it could also reflect the signal detection properties of the test itself, as we explain below. Thus, it is premature to make strong claims about the diversity/heterogeneity of the emotion categories based on these results.

Heterogeneity in the representation across categories occurs both because some of the methods used to elicit emotion are more diverse (S2 Table) and because the categories are likely to be inherently psychologically and neurally diverse.

We think of each emotion category as a population of diverse instances, rather than a homogeneous set of instances. Thus, there may be multiple types of ‘anger’ that activate different subsets of regions and networks. What we observe is the population average across these potentially disparate features. This is analogous to dwellings containing disparate architectural features (e.g., an igloo vs. a castle) being grouped into a common category (‘dwelling’) because of their cultural functions rather than their architectures. On a more mundane level, the ways in which researchers choose to study emotion categories can also contribute to the observed diversity (and reduced classification accuracy); researchers studying fear, for example, tend to sample very similar instances by using a small range of fear-inducing methods, whereas anger is elicited in more diverse ways.

A second potential reason for differences and accuracy relates to the signal detection properties of the model. Sensitivity and specificity can always be traded off by changing the decision threshold for labeling an instance as ‘anger,’ ‘fear,’ etc., and accuracy in 5-way classification is more closely related to sensitivity than specificity. Here, anger has the lowest sensitivity (43%), but the highest specificity (99%, Table 1): Studies that are not anger are almost never categorized as anger. Such differences in threshold preclude making strong claims about the diversity/heterogeneity of the emotion categories themselves based on these results. However, we should not be too quick to dismiss all differences in decoding accuracy to methodological artifacts; true differences in category heterogeneity may exist as well.

### Limitations

A number of important issues and limitations remain to be addressed. First, our analyses reflect the composition of the studies available in the literature, and are subject to testing and reporting biases on the part of authors. This is particularly true for the amygdala (e.g., the activation intensity for negative emotions may be over-represented in the amygdala given the theoretical focus on fear and related negative states). However, the separation of emotion categories in the amygdala was largely redundant with information contained in cortical patterns, which may not be subject to the same biases. Likewise, other interesting distinctions were encoded in the thalamus and cerebellum, which have not received the theoretical attention that the amygdala has and are likely to be bias-free.

Secondly, these results are limited by the inherent resolution and signal properties of the original studies. Some regions—particularly the brainstem—are likely to be much more important for understanding and diagnosing emotion than is apparent in our findings, because neuroimaging methods are only now beginning to focus on the brainstem with sufficient spatial resolution and artifact-suppression techniques (Satpute et al., 2013). Other areas that are likely to be important, such as the ventromedial prefrontal cortex (e.g., BA 25 and posterior portions of medial OFC) are subject to signal loss and distortion, and are likely to be under-represented.

Thirdly, there is always the possibility that differences in study procedures or the involvement of processes not directly related to emotional experience could partially explain some findings. A meta-analytic result is only as good as the data from which it is derived, and a brief look at S1 Fig indicates that there are some systematic differences in the ways researchers have studied (and evoked instances of) different emotion categories. We have tried to systematically assess the influence of methodology differences in this paper, but our ability to do this is imperfect. However, though we cannot rule out all possible methodological differences, we should not be too quick to dismiss findings in ‘sensory processing’ areas, etc., as methodological artifacts. Emotional responses may be inherently linked to changes in sensory and motor cortical processes that contribute to the emotional response (e.g., [101]). This is a central feature of both early and modern embodiment-based theories of emotion [92,102–104]. In addition, most major theories of emotion suggest that there are systematic differences in cognitive, perceptual, and motor processes across emotion categories; and in some theories, such as the appraisal theories, those differences are inherently linked to or part of the emotional response [105].

Finally, the results we present here provide a co-activation based view of emotion representation that can inform models of functional connectivity. However, co-activation is not the same as functional connectivity. The gold-standard measures of direct neural connectivity use multiple single-unit recording or optogenetics combined with single-unit electrophysiology to identify direct neural connections with appropriate latencies (e.g., < 20 msec). Much of the information processing in the brain that creates co-activation may not relate to direct neural connectivity at all, but rather to diffuse modulatory actions (e.g., dopamine and neuropeptide release, much of which is extrasynaptic and results in volume transmission). Thus, the present results do not imply direct neural connectivity, and may be related to diffuse neuromodulatory actions as well as direct neural communication. However, these forms of brain information processing may be important in their own right.

## Methods

### Database

The dataset consists of activation foci from 397 fMRI and PET studies of emotion published between 1990 and 2011, which included a total of 914 unique study activation maps and 6,827 participants. Activation foci are coordinates reported in Montreal Neurologic Institute standard anatomical space (or transformed from Talairach space). Foci are nested within study activation maps, maps of group comparisons between an emotion- or affect-related condition and a less intense or affectively neutral comparison condition. We used the foci associated with study activation maps to predict each map’s associated emotion category. Studies were all peer-reviewed and were identified in journal databases (PubMed, Google Scholar, and MEDLINE) and in reference lists from other studies. A subset of studies that focused specifically on the most frequently studied emotion categories were selected (148 studies, 377 maps, 2519 participants). Categories included anger (69 maps), disgust (69 maps), fear (97 maps), happiness (77 maps), and sadness (65 maps).

### Bayesian Spatial Point Processes (BSPP) for Neuroimaging Meta-Analysis

The BSPP is built on a hierarchical marked independent cluster process designed for functional neuroimaging meta-analysis [38]. We model the foci (peak activation locations) as the offspring of a latent study center process associated with a study activation map. The study centers are in turn offspring of a latent population center process. The posterior intensity function of the population center process provides inference on the location of population centers, as well as the inter-study variability of foci about the population centers.

Specifically, the model has three levels of hierarchy. At level 1, for each study, we assume the foci are a realization of an independent cluster process driven by a random intensity function. These processes are independent across studies. The study level foci are made up of two types of foci: singly reported foci and multiply reported foci. For a given activation area in the brain, some authors only report a single focus, while others report multiple foci, however this information is rarely provided in the literature. These differences are attributable to how different software packages report results, and simply author preference. We assume that multiply reported foci cluster about a latent study activation center, while the singly reported foci can either cluster about a latent population center or are uniformly distributed in the brain. At level 2, we model the latent study activation center process as an independent cluster process. We assume that the latent study activation centers can either cluster about the latent population center or are uniformly distributed in the brain. At level 3, we model the latent population center process driven by a homogeneous random intensity (a homogeneous Poisson process). The points that may cluster about the population centers are singly reported foci from level 1 and study activation centers from level 2.

We make inference on the latent population centers in a Bayesian framework. In particular, we use spatial birth and death processes nested within a Markov chain Monte Carlo simulation algorithm. The details of the algorithm and pseudo code are provided in [38].

### Bayesian Spatial Point Process Classification Model

The BSPP model estimates the posterior distribution for reported foci across studies. Foci reported in each emotion category can be modeled as an emotion-specific spatial point process. This leads to a joint model for foci from studies with different categories of emotions, and it can be used to classify the emotion category of studies given their observed foci, by choosing the emotion category that maximizes the posterior predictive probability. To be more specific, suppose we have *n* studies, let *F_i_* and *E_i_* denote the foci and the emotion category for study *i* respectively, for *i* = 1, …, *n*. The BSPP model specifies the probability π (*F_i_*|*E_i_*, *λ*), where *λ* represents the collection of all the parameters in the BSPP model. The posterior predictive probability of emotion category for a new study *E*_*n*+1_ is given by $\text{Pr}(E_{n+1}\;=\;e\;|\;{\left(F_{i},\;E_{i}\right)}_{i=1}^{n},\;F_{n+1})\;\propto \;\text{Pr}(E_{n+1}\;=\;e)\;\int \;\Pi_{i=1}^{n}\;\pi \;(F_{i}\;|\;E_{i},\;\lambda )\;\pi \;(F_{n+1}\;|\;E_{n+1}\;=\;e,\;\lambda )\;\pi \;(\lambda )\;d\lambda$, for *e* = 1, …, *m*, where *m* represents the total number of emotion categories. Pr(*E*_*n*+1_ = *e*) represents the prior probability of emotion category for the study type and *π*(*λ*) is the prior of parameters.

The performance of the proposed classifier is evaluated via leave-one-out cross validation (LOOCV) on all the observed data, i.e., leaving one study out. We conduct Bayesian learning of the model parameters on the foci reported from a set of training studies consisting of all studies except a left-out study, *k*. We then make a prediction for study *k* based on its reported brain foci. We repeat the procedure for each study (1…*K*) and compute the classification rate across all studies. The above procedure for a Bayesian model can be very computationally expensive since it involves multiple posterior simulations. We employ an importance sampling method to substantially reduce the computation. See [106] for details.

### Emotional Signatures Across Networks and Regions of Interest

To investigate the similarities and differences among emotion categories in defined resting-state fMRI and anatomical networks, we identified a priori networks and regions from published studies as described above [42–44, 107, 108] (see also Fig. 2 legend). These networks covered the entire cerebrum, excluding white matter and ventricles. Within each of the 49 regions, we calculated the average BSPP intensity value across voxels for each emotion category. Analyses of the mean intensity across regions/networks are visualized in Figs. 2 and S3 and S1 Text.

#### Calculation of region/network mean intensity

For Markov chain Monte Carlo (MCMC) iterations *t* = [1…*T*] (T = 10,000 in this analysis), region/network *r* = [1…*R*], and emotion categories *c* = [1…*C*] (C = 5 in this analysis), let *M*_*c*_ be a *T* x *R* matrix of mean intensity values in each region for each iteration for emotion *c*. We calculated the mean intensity ‘signature across regions’ for each emotion category, ${\vec{M}}_{c}\;=\;\left[\frac{\sum_{t=1}^{T}M_{ct1}}{T}\;\;\;\;\frac{\sum_{t=1}^{T}M_{ct2}}{T}\cdots \frac{\sum_{t=1}^{T}M_{ctR}}{T}\right]$, which are shown for subsets of regions in Figs. 2 and S3. In addition, the matrix *M*_*c*_ contains samples from the joint posterior distribution of regional intensity values that can be used for visualization and statistical inference. Mean intensity values for each region/network served as nodes in co-activation analyses.

#### Co-activation and graphical representation

Visualizations of the configuration of regions associated with each emotion (Figs. 4, S4) was performed by estimating the pairwise correlations among all regions *r* = [1…*R*]across MCMC iterations (e.g., for regions i and j, the correlation between *M*_c_ •*i*and *M*_c_ •*j*). Thresholded correlations served as edges in co-activation analyses. The correlations were thresholded using a permutation test, as follows: We permuted the rows of each vector *M*_c_ •*r*, for *r* = [1…*R*], independently, and calculated the maximum correlation coefficient across the R x R correlation matrix for each of 1000 iterations. The 95th percentile of this distribution was used as the threshold, which controlled for matrix-wise false positives at P <. 05 family-wise error rate corrected. The most stringent threshold across emotion category (r > 0.0945, for sadness) was used for all emotion categories, to maintain a consistent threshold across graphs. The location of each node (region/network) on the graph was determined by applying the Fruchterman-Reingold force-directed layout algorithm (as implemented in the MatlabBGL toolbox by David Gleich) to the thresholded correlation matrix.

#### Statistical inference on activation ‘signatures across regions.’

The MCMC sampling scheme also provided a means of making statistical inferences on whether the multivariate ‘pattern of regional’ of intensity differed across pairs of emotion category (S1 Table). 1). For any given pair of emotion categories *i* and *j*, the difference between the intensity fingerprints is given by the vector ${\vec{M}}_{d}\;=\;{\vec{[M}}_{i}\;-\;{\vec{M}}_{j}]$. As the elements of M are samples from the joint posterior distribution of intensity values, statistical inference on the difference ${\vec{M}}_{d}$ depends on its statistical distance from the origin, which is assessed by examining the proportion *P* of the samples that lie on the opposite side of the origin from ${\vec{M}}_{d}$, adjusting for the fact that the mean ${\vec{M}}_{d}$ could occur in any of the 2^R^ quadrants of the space defined by the regions. This is given by: $P\;=\;\frac{2^{R}}{T}\Sigma_{t=1}^{T}\Sigma_{r=1}^{R}\;\mathrm{abs}(\mathrm{sign}(M_{\mathrm{itr}}\;-\;M_{\mathrm{jtr}})\;-\;\mathrm{sign}(\vec{M_{d}{}_{{}_{r}}}))\;\geq \;R$. This corresponds to a nonparametric P-value for the difference in posterior intensity profiles across regions from the MCMC algorithm, which is subjected to false discovery rate control across the $\frac{C(C-1)}{2}$ pairwise comparisons at *q* <. 05. It is an analogue to the multivariate F-test in parametric statistics. This test can be conducted across profiles within a set of regions/networks (e.g., cortical networks shown in Fig 4A), across all regions, or across the intensity scores in non-negative components of activation ‘patterns across regions.’

### Non-negative Matrix Factorization

Non-negative matrix factorization (NNMF) is a way of decomposing a complex data set into simpler, additive components that are particularly interpretable [109]. Here, we used it to decompose the matrix of activation intensities for each of the five emotions across subgroups of 49 regions into simpler, additive ‘profiles’ of activation shown in polar plots in Figs. 2 and S3. The activation matrix A is decomposed into two component matrices *W*(*n*•*k*) and *H*(*k*•*m*) whose elements are non-negative, such that *A* = *WH*, with the number of components (*k*) selected a priori (here, *k* = 2 for interpretability and visualization). The squared error between A and WH was minimized via an alternating least squares algorithm with multiple starting points. The rows of H constitute the canonical profiles shown in figures, and emotion-specific activation intensity values from the BSPP model are plotted in the 2-dimensional space of the two recovered canonical activation profiles.

NNMF is a particularly appropriate and useful decomposition technique here, because activation intensity is intrinsically non-negative [110,111]. In such cases, NNMF identifies components that are more compact and interpretable than principal components analysis (PCA) or independent components analysis (ICA), and better reflect human intuitions about identifying parts that can be additively combined into wholes. Here, the parts reflect interpretable, canonical activation profiles, and the whole is the observed activation profile for each emotion category across multiple brain systems.

## Supporting Information

S1 Text Support Vector Machine analyses. The Bayesian Spatial Point Process Model classification results are compared against the support vector machine-based classification described here.
(PDF)

S1 Table Classification accuracy tables and confusion matrices for five-way emotion classification for several methods. Results from the Bayesian Spatial Point Process Model [38,106] are the focus of this paper, and other methods are included for comparison purposes. Row labels reflect the true category, and column labels the predicted category. Diagonals (red) indicate accuracy or recall proportions. Off-diagonals indicate error proportions. *: Accuracy is significantly above chance based on a binomial test.
(PDF)

S2 Table A summary of statistical tests on co-activation patterns in the network analysis. (PDF)

S3 Table Emotion classification based on methodological variables. (PDF)

S1 Fig Information about methodological variables in the studies. A) Map of relationships between emotion categories and methodological variables, including emotion elicitation method, stimulus type used, participant sex, and imaging technique. Colors represent proportions of studies in a given emotion category that involved each method variable; columns each sum to 1 (100% of studies) within each method variable. Ideally, stimulus category and other methodological variables would be evenly distributed across the five emotion categories (i.e., each row would be approximately evenly colored across emotion categories), although this is impractical in practice, and the distribution depends on how investigators have chosen to conduct studies. See S2 Table for additional information about classification of emotion type from these methodological variables.
(PDF)

S2 Fig Images of each of the five emotion category intensity maps across the whole brain. Intensity maps for each of the five emotion categories. Intensity maps reflect the distribution of study activation centers (Level 2 in the Bayesian model) across brain space. They are continuously valued across space, though they are sampled in voxels (2 x 2 x 2 mm), and the integral of the intensity map over any area of space reflects the expected number of study-level centers for that emotion category. Brighter colors indicate higher intensity, and the maps are thresholded at a value of 0.001 for display.
(PDF)

S3 Fig Intensity profiles and non-negative matrix factorizations for subcortical structures. Subcortical zones and activation intensity profiles within each. A) Left: Basal ganglia regions of interest based on the Buckner Lab’s 1000-person resting state connectivity analyses (9–11), along with amygdala and parahippocampal/hippocampal regions of interest based on the probabilistic atlas of Amunts et al. (12) (see Main Text). Network labels follow the conventions used in the Buckner Lab papers. Right: Intensity maps for each emotion category displayed on surface images of the basal ganglia, amygdala, and hippocampus. B) Intensity profiles for each emotion category (colored lines, colors as in (A)) in subcortical zones. Values towards the perimeter of the circle indicate higher activation intensity. In the amygdala, LB: basolateral complex; CM: corticomedial division; SF: superficial division. L: left; R: right hemisphere. In the hippocampus: FD, dentate; CA, Cornu Ammonis; SUB, subicular zone. C) Intensity distribution for each emotion (colors) in the space of the first two factors from non-negative matrix factorization of the intensity profiles. The colors correspond to the emotion category labels in (A), and the colored areas show the 95% confidence region for the activation intensity profiles.
(PDF)

S4 Fig Network topology graphs with labels for all nodes. Connectivity graphs for each emotion category, as in Fig. 3, but with labels for all regions/networks. A) anger; B) disgust; C) fear; D) happy; E) sad. The layouts are force-directed graphs for each emotion category, based on the Fruchterman-Reingold spring algorithm. The nodes (circles) are regions or networks, color-coded by anatomical system. The edges (lines) reflect co-activation between pairs of regions or networks, assessed based on the joint distribution of activation intensity in the Bayesian model at P <. 05 corrected based on a permutation test. The size of each circle reflects its betweenness-centrality (ref), a measure of how strongly it connects disparate networks. Region labels are as in Figs. 2 and S3. Colors: Cortex, red; Basal ganglia, green; Cerebellum/brainstem, blue; Thalamus, yellow; Amygdala, magenta; Hippocampus, cyan/light blue. Network names follow the convention used in the Bucker Lab’s resting-state connectivity papers: V, visual network/zone (in cortex and connected regions in basal ganglia and cerebellum); dA, dorsal attention; vA, ventral attention; FP, fronto-parietal; S, somatosensory; DM, default mode; L, limbic. Thalamic regions are from the connectivity-based atlas of Behrens et al. (13). Tem, temporal; Som, somatosensory; Mot, motor; Pmot, premotor; Occ, occipital, PFC, prefrontal cortex connectivity. Other regions: Hy, hypothalamus; B (yellow letter on blue node), brainstem.
(JPEG)

S5 Fig Average correlation values within and between region groups, and relationship with global network efficiency and regional activation intensity. Average co-activation within and between each region/network grouping, for comparison to global network efficiency values based on path length in Fig. 3. Top: Average correlation in regional intensity across 10,000 MCMC samples in the Bayesian model. These correlations provide a measure of co-activation across disparate brain networks. The overall pattern is similar to Fig. 3; however, the average correlation does not reflect some of the structure captured in global efficiency and reflected in the graphs in Fig. 3. Bottom left: Average correlation is related to global efficiency across network groups and emotion categories (r = 0.76). Each point reflects an element of the matrices in the top panel. Bottom right: Global efficiency is unrelated to average activation intensity within the regions being correlated (r = 0.02), indicating that the efficiency metric used in the main manuscript provides information independent of the marginal activation intensity.
(PDF)
