# Supplementary material for: A Bayesian Model of Category-Specific Emotional Brain Responses
Source: PLoS Comput Biol. 2015 Apr 8;11(4):e1004066. doi: 10.1371/journal.pcbi.1004066 (PMC4390279; doi:10.1371/journal.pcbi.1004066)
Supplement: S1 Text — The Bayesian Spatial Point Process Model classification results are compared against the support vector machine-based classification described here. (PDF) [file pcbi.1004066.s001.pdf]

A Bayesian model of category-specific emotional brain responses

SUPPLEMENTARY INFORMATION

Tor D. Wager[1]
Jian Kang[2]
Timothy D. Johnson[3]
Thomas E. Nichols[4,5]
Ajay B. Satpute[6]
Lisa Feldman Barrett[6,7]

[1] Department of Psychology and Neuroscience and the Institute for Cognitive Science, University of Colorado, Boulder
[2] Department of Biostatistics and Bioinformatics, Department of Radiology and Imaging Sciences, Emory University
[3] Department of Biostatistics, University of Michigan
[4] Department of Statistics and Warwick Manufacturing Group, University of Warwick, UK
[5] Functional Magnetic Resonance Imaging of the Brain (FMRIB) Centre, Nuffield Department of Clinical Neurosciences, University of Oxford, UK
[6] Department of Psychology, Northeastern University
[7] Massachusetts General Hospital/Harvard Medical School

Running Head: DECODING EMOTION CATEGORIES - SUPPLEMENT

Please address correspondence to:

Tor D. Wager
Department of Psychology and Neuroscience
University of Colorado, Boulder
345 UCB
Boulder, CO 80309
Email: tor.wager@colorado.edu
Telephone: (303) 895-8739

Supplementary methods

Support vector machine (SVM) analysis

We performed a ‘one-vs-one’ classification analysis of the five emotion categories using a series of nonlinear SVM analyses (e.g., (1)) with parameters chosen a priori (slack parameter C = 1, radial basis function kernel with standard deviation = 15 mm) as implemented in the Spider machine learning toolbox for Matlab. We used stratified 10-fold cross-validation to assess accuracy in classifying the emotion categorie associated with each study activation map. In each fold, approximately 90% of the maps were used to train the SVM algorithm (‘training set’), and the remaining 10% were used to assess classification accuracy (‘test set’). For each fold, we selected maps from the training set associated with each pairwise comparison of emotion categories (e.g., anger vs. sadness, etc.), and trained an SVM to discriminate that pair (i.e., 10 separate SVMs were trained for each fold). In each SVM, foci from the training set were individually labeled with the associated activation map’s emotion category and used to identify a separating hyperplane; combined with the radial kernel, this produces regions of the brain discriminative of one emotion category vs. the other member of each pair. To predict the emotion category on the test set, foci from each test-set study map were aggregated by averaging the distance from the SVM hyperplane across foci, to derive a single predicted class for each map. Then, information across the separate pairwise SVMs was aggregated using a voting method, with the map’s final predicted class being the emotion category with the most votes.

Bayesian spatial point processes (BSPP)

Model Assumptions

1. Study level foci are realizations of independent cluster processes, i.e., instances drawn from a population with spatial coordinates surrounding a set of fixed population centers (i.e., ‘true’ activation centers).
2. The intensity functions of all the latent independent cluster processes in the model take the form of mixtures of Gaussian kernels. In other words, all the point pattern clusters in the model have a Gaussian shape, though the parameters of those Gaussians may vary.
3. For a given activation area in the brain, some studies only report a single focus, while others report multiple foci. The number of study foci reported is unknown in the model and is estimated, given reasonable prior assumptions.
4. Some estimates based on prior knowledge is needed for posterior inference in the Bayesian framework: the expected number of population centers, the expected number of multiply reported peaks per study, and the expected number of activation centers cluster about population centers.

Notation

Denote by $PP(\beta)$ a homogeneous Poisson point process on brain region B with intensity β. Denote by $ICP(\epsilon, \theta, \mathbf{u}, \mathbf{\Omega})$ an independent cluster process on brain region B driven by random intensity function $\lambda(x \mid \epsilon, \theta, \mathbf{u}, \mathbf{\Omega}) = \epsilon + \sum_{(u,\Omega_u)\in(\mathbf{u},\mathbf{\Omega})} \theta\phi_3(x; u, \Omega_u)$, where $\phi_3(x; u, \Omega_u)$ represents a three-dimensional Gaussian density function with mean u and covariance Ω_u; $(\mathbf{u}, \mathbf{\Omega})$ is a marked latent point process with $u \in \mathbf{u}$ representing the location of the cluster center and the mark $\Omega_u \in \mathbf{\Omega}$ charactering the shape of the cluster; the parameter ϵ represents the intensity of a latent homogeneous Poisson process of “background noise level”; and θ controls the expected number of points clustering about each center. Denote by X_c foci (data) for study c, $c = 1, \dots, C$, The study level foci are made up of two types of foci: singly reported foci (Type 0 foci) and multiply reported foci (Type 1 foci), denoted X_c^0 and X_c^1, respectively. Denote by (Y_c, Ψ_c) the marked latent activation center process for study c and (Z, Σ) the marked latent population center process.

Model Description

The BSPP model involves three levels of hierarchy:

At level 1, we assume that multiply reported foci cluster about a latent study activation center, while the singly reported foci can either cluster about a latent population center, or are uniformly distributed in the brain. That is

$$X_c^0 \mid \epsilon_{1c}, \theta_{1c}, Z, \Sigma \sim ICP(\epsilon_{1c}, \theta_{1c}, Z, \Sigma)$$

$$X_c^0 \mid \eta_c, Y_c, \Psi_c \sim ICP(0, \eta_c, Y_c, \Psi_c)$$

where θ_{1c} controls the expected number of Type 0 foci that cluster about a population center; parameter ϵ_{1c} is the intensity of the Type 0 foci that do not cluster about a population center; parameter η_c controls the expected number of Type 1 foci that cluster about a study activation center.

At level 2, we assume that the latent study activation centers can either cluster about the latent population center or are uniformly distributed in the brain. That is

$$Y_c \mid \epsilon_{2c}, \theta_{2c}, Z, \Sigma \sim ICP(\epsilon_{2c}, \theta_{2c}, Z, \Sigma)$$

where θ_{2c} controls the expected number of Type 1 foci that cluster about a population center; parameter ϵ_{2c} is the intensity of the Type 1 foci that do not cluster about a population center;

At level 3, we assume that the latent population center process driven by a homogeneous random intensity (a homogeneous Poisson process).

$$Z \mid \beta \sim PP(\beta)$$

where β controls the expected number of population centers.

BSPP classification model

Suppose we consider m emotion category. Let $E_c \in \{1, \ldots, m\}$ denote the emotion category for study c. For all the studies of emotion category $e, e = 1, \ldots, m,$ we can fit the

BSPP model to make inference on the probability $\pi(X_c \mid E_c = e, \Theta_e)$, where Θ_e represents a collection of all the parameters in the BSPP model for emotion e. Denote by $\Theta = (\Theta_1, \ldots, \Theta_m)$ all the parameters across different emotions. The posterior predictive probability of emotion category for a new study with foci X_{new} is given by

$$\Pr\left(E_{new} = e \mid (X_c, E_c)_{i=1}^{C}, X_{new}\right) \propto$$

$$\Pr(E_{new} = e) \int \prod_{i=1}^{C} \pi\left(X_c \mid E_c, \Theta\right) \pi\left(X_{new} \mid E_{new} = e, \Theta\right) \pi(\Theta)\, d\Theta,$$

for $e = 1, \ldots, m$, where $\Pr(E_{new} = e)$ represents the prior probability of emotion category e and $\pi(\Theta)$ is the prior of parameters.

Permutation Test

We performed a permutation test to validate classification results under the null hypothesis that the emotion categories are independent of the study foci in order to confirm the BSSP based classification model did not over-fit the data. Specifically, we permuted the emotion labels of studies and created 100 permuted datasets. Then we applied the BSSP classification model for each permuted dataset and obtained the null distribution of the confusion matrix. We summarize the mean, standard deviation (Sd) and lower (LCL) and upper (UCL) 95% confidence intervals in the table below.

For the mean, which reflects overall classification accuracy in permuted data, the expected value is 0.2 if the test is unbiased. As the Table shows, all tests were unbiased, with 0.2 well within the margin of error based on the number of permutations. The upper confidence bound for accuracy in any condition was approximately 0.3, so values above this in actual classification may be considered significant at $p < .05$ family-wise error rate corrected across categories.

Permutation test: Null hypothesis classification table

Mean	Anger	Disgust	Fear	Happy	Sad
Anger	0.195	0.201	0.208	0.19	0.206
Disgust	0.202	0.194	0.2	0.199	0.204
Fear	0.193	0.196	0.207	0.204	0.199
Happy	0.201	0.204	0.202	0.196	0.197
Sad	0.204	0.2	0.193	0.197	0.207

Sd	Anger	Disgust	Fear	Happy	Sad
Anger	0.039	0.04	0.042	0.039	0.043
Disgust	0.045	0.046	0.049	0.045	0.046
Fear	0.04	0.04	0.046	0.046	0.044
Happy	0.04	0.046	0.048	0.042	0.043
Sad	0.041	0.042	0.04	0.046	0.045

UCL	Anger	Disgust	Fear	Happy	Sad
Anger	0.261	0.269	0.278	0.265	0.295
Disgust	0.304	0.286	0.298	0.295	0.296
Fear	0.264	0.267	0.302	0.303	0.283
Happy	0.286	0.297	0.299	0.279	0.3
Sad	0.273	0.267	0.274	0.282	0.292

LCL	Anger	Disgust	Fear	Happy	Sad
Anger	0.119	0.132	0.139	0.131	0.129
Disgust	0.128	0.112	0.115	0.117	0.125
Fear	0.098	0.124	0.108	0.13	0.126
Happy	0.122	0.119	0.122	0.123	0.125
Sad	0.128	0.131	0.115	0.112	0.121

Non-negative matrix factorization

Non-negative matrix factorization (NNMF) is a way of decomposing a complex data set into simpler, additive components. It is similar to principal components analysis (PCA) and independent components analysis (ICA) in this respect, but with a major advantage: The non-negativity constraint causes the recovery of hidden features (components) that

are more compact and interpretable than PCA or ICA ((2). It is particularly appropriate for cases in which the data distribution is non-negative; for example, in the coordinate-based meta-analysis data we analyze here, study activation counts (and thus density) can never be negative. Thus, meta-analysis is a natural application, and previous approaches have used NNMF successfully (3, 4)

NNMF decomposes the n-by-m matrix A, here an [n x 5] matrix of average intensity for 5 emotions (m = 5) in each of n regions or networks, into two component matrices W (n x k) and H (k x m) whose elements are non-negative, such that A = WH. k is the number of components retained, usually up to nm / (n + m) components. The component matrices are chosen such that WH most closely approximates the original matrix A (i.e., with minimal error variance). This additive decomposition permits the combination of multiple basis vectors (here, profiles of activation intensity across regions) to represent an emotion category. Because interpretability is an explicit goal, we decomposed profiles of activity across emotion types (e.g., n x 5 matrices, where n is the number of networks in cortex, basal ganglia, or cerebellum, or regions in amygdala, hippocampus, or thalamus) into two components (k = 2), so that emotion-specific activation intensity values could be plotted in the 2-dimensional space of the two canonical activation profiles.

The non-negativity constraint provides a natural way of increasing the interpretability of the resulting component vectors (or profiles across regions/networks). PCA eigenvectors usually involve complex cancellations between positive and negative loadings on regions/networks, making the components hard to interpret individually. NNMF, by contrast, recovers solutions that are more compatible with human intuitions about how to interpret patterns. The components represent parts that can be combined

additively into a whole (2, 5) Here, the "parts" are canonical activation profiles across individual regions/networks, which can be combined additively into a model of the profile of activation intensity for a given emotion.

The implementation of NNMF we used (Matlab R2013b) uses an iterative alternating least squares method to optimize W and H (see (6)). It starts with random initial values for W and H; because the solution can sometimes vary across starting points, we ran all NNMF analyses with 100 replications and averaged their values. The algorithm never converged on solutions of less than two components.

Once the two canonical profiles were obtained for each grouping of regions/networks (e.g., cortex, thalamus, etc.), the profiles of observed activation intensity for each emotion (ym) were regressed on the component values (e.g., ym ~ H'), yielding an overall expression (slope) of the two canonical profiles for that emotion. These regressions were performed for all 10,000 MCMC iterations, yielding a posterior loading distribution for each emotion in the canonical NNMF profile space. These are plotted in Figure 2C (for cortex) and Supplementary Figure 3C (for other groups). The axes of these plots represent canonical profiles discovered across all emotions (i.e., the two rows of H), and the posterior density for each emotion is depicted in an emotion-specific color, with the lightest shading approximately at the 95% credibility region boundary for the emotion. The plots thus show how different the emotions are in some cases in the canonical profile space defined by NNMF.

References

1. Vapnik VN (1999) An overview of statistical learning theory. *IEEE Trans Neural Netw* 10(5):988-999.
2. Lee DD & Seung HS (1999) Learning the parts of objects by non-negative matrix factorization. *Nature* 401(6755):788-791.
3. Lee JH, Hashimoto R, Wible CG, & Yoo SS (2011) Investigation of spectrally coherent resting-state networks using non-negative matrix factorization for functional MRI data. *International Journal of Imaging Systems and Technology* 21(2):211-222.
4. Nielsen FÅ, Hansen LK, & Balslev D (2004) Mining for associations between text and brain activation in a functional neuroimaging database. *Neuroinformatics* 2(4):369-379.
5. Donoho D & Stodden V (2003) When does non-negative matrix factorization give a correct decomposition into parts? *Advances in neural information processing systems*, p None.
6. Lee DD & Seung HS (2001) Algorithms for non-negative matrix factorization. *Advances in neural information processing systems*, pp 556-562.
7. Kang J (2011) Some Novel Spatial Stochastic Models for Functional Neuroimaging Analysis. Ph.D. (University of Michigan, Ann Arbor).
8. Kang J, Johnson TD, Nichols TE, & Wager TD (2011) Meta analysis of functional neuroimaging data via Bayesian spatial point processes. *Journal of the American Statistical Association* 106(493):124-134.
9. Buckner RL, Krienen FM, Castellanos A, Diaz JC, & Yeo BT (2011) The organization of the human cerebellum estimated by intrinsic functional connectivity. *Journal of Neurophysiology* 106(5):2322-2345.
10. Yeo BT*, et al.* (2011) The organization of the human cerebral cortex estimated by intrinsic functional connectivity. *J Neurophysiol* 106(3):1125-1165.
11. Choi EY, Yeo BT, & Buckner RL (2012) The organization of the human striatum estimated by intrinsic functional connectivity. *Journal of Neurophysiology* 108(8):2242-2263.
12. Amunts K*, et al.* (2005) Cytoarchitectonic mapping of the human amygdala, hippocampal region and entorhinal cortex: intersubject variability and probability maps. *Anatomy and Embryology* 210(5-6):343-352.
13. Behrens T*, et al.* (2003) Non-invasive mapping of connections between human thalamus and cortex using diffusion imaging. *Nature Neuroscience* 6(7):750-757.
