# Supplementary material for: A Bayesian Model of Category-Specific Emotional Brain Responses
Source: PLoS Comput Biol. 2015 Apr 8;11(4):e1004066. doi: 10.1371/journal.pcbi.1004066 (PMC4390279; doi:10.1371/journal.pcbi.1004066)
Supplement: S1 Table — Results from the Bayesian Spatial Point Process Model [38,106] are the focus of this paper, and other methods are included for comparison purposes. Row labels reflect the true category, and column labels the predicted category. Diagonals (red) indicate accuracy or recall proportions. Off-diagonals indicate error proportions. *: Accuracy is significantly above chance based on a binomial test. (PDF) [file pcbi.1004066.s002.pdf]

Table S1: Confusion matrices

Bayesian Spatial Point Process Model

	Anger	Disgust	Fear	Happy	Sad
Anger	**0.43***	0.07	0.28	0.15	0.08
Disgust	0.03	**0.76***	0.08	0.08	0.06
Fear	0.02	0.04	**0.86***	0.06	0.03
Happy	0.00	0.07	0.23	**0.58***	0.11
Sad	0.00	0.07	0.20	0.09	**0.65***

Average accuracy is 0.66

Naïve Bayes Classifier

	Anger	Disgust	Fear	Happy	Sad
Anger	**0.17**	0.11	0.51	0.12	0.09
Disgust	0.13	**0.37***	0.36	0.05	0.09
Fear	0.05	0.09	**0.73***	0.04	0.08
Happy	0.09	0.12	0.43	**0.23**	0.13
Sad	0.11	0.11	0.42	0.10	**0.25**

Average accuracy is 0.35

Support vector machine classifier

	Anger	Disgust	Fear	Happy	Sad
Anger	**0.28**	0.14	0.28	0.19	0.12
Disgust	0.16	**0.32***	0.17	0.16	0.19
Fear	0.13	0.09	**0.51***	0.12	0.14
Happy	0.18	0.16	0.26	**0.23**	0.17
Sad	0.23	0.11	0.22	0.14	**0.31**

Average accuracy is 0.33

Bayesian Spatial Point Process Model controlling for Induction Method

	Anger	Disgust	Fear	Happy	Sad
Anger	**0.40***	0.11	0.29	0.13	0.07
Disgust	0.03	**0.72***	0.08	0.09	0.09
Fear	0.03	0.03	**0.83***	0.07	0.04
Happy	0.00	0.13	0.23	**0.49***	0.15
Sad	0.00	0.07	0.23	0.09	**0.62***

Average accuracy is 0.61
