# Supplementary material for: A Bayesian Model of Category-Specific Emotional Brain Responses
Source: PLoS Comput Biol. 2015 Apr 8;11(4):e1004066. doi: 10.1371/journal.pcbi.1004066 (PMC4390279; doi:10.1371/journal.pcbi.1004066)
Supplement: S2 Table — (PDF) [file pcbi.1004066.s003.pdf]

Supplementary Table S2. Emotion classification based on methodological variables

Emotion elicitation method

	Anger	Disgust	Fear	Sad	Happy
**Anger**	**0.01**	0.00	0.70	0.29	0.00
**Disgust**	0.04	**0.00**	0.83	0.13	0.00
**Fear**	0.00	0.00	**0.90**	0.10	0.00
**Sad**	0.09	0.02	0.49	**0.29**	0.11
**Happy**	0.00	0.00	0.70	0.30	**0.00**
**Average accuracy:**		**0.24 n.s.**			

Imaging technique (PET or fMRI)

	Anger	Disgust	Fear	Sad	Happy
**Anger**	**0.00**	0.78	0.00	0.22	0.00
**Disgust**	0.00	**0.94**	0.00	0.06	0.00
**Fear**	0.00	0.94	**0.00**	0.06	0.00
**Sad**	0.00	0.63	0.00	**0.37**	0.00
**Happy**	0.00	0.73	0.00	0.27	**0.00**
**Average accuracy:**		**0.26 p < .05**			

Stimulus type

	Anger	Disgust	Fear	Sad	Happy
**Anger**	**0.06**	0.04	0.65	0.25	0.00
**Disgust**	0.07	**0.33**	0.42	0.17	0.00
**Fear**	0.05	0.15	**0.68**	0.07	0.04
**Sad**	0.02	0.11	0.29	**0.51**	0.08
**Happy**	0.00	0.05	0.62	0.32	**0.00**
**Average accuracy:**		**0.32 p < .05**			

Participant sex

	Anger	Disgust	Fear	Sad	Happy
**Anger**	**0.83**	0.00	0.13	0.04	0.00
**Disgust**	0.55	**0.00**	0.16	0.29	0.00
**Fear**	0.69	0.00	**0.23**	0.08	0.00
**Sad**	0.55	0.29	0.15	**0.00**	0.00
**Happy**	0.66	0.00	0.17	0.17	**0.00**
**Average accuracy:**		**0.21 n.s.**			

Task Type

	Anger	Disgust	Fear	Sad	Happy
**Anger**	**0.00**	0.29	0.32	0.09	0.30
**Disgust**	0.00	**0.49**	0.28	0.07	0.16
**Fear**	0.00	0.29	**0.44**	0.02	0.25
**Sad**	0.00	0.29	0.17	**0.38**	0.15
**Happy**	0.31	0.32	0.21	0.16	**0.00**
**Average accuracy:**		**0.26 p < .05**			

Comparison (control) condition

	Anger	Disgust	Fear	Sad	Happy
**Anger**	**0.00**	0.20	0.70	0.06	0.04
**Disgust**	0.00	**0.00**	0.68	0.00	0.32
**Fear**	0.00	0.20	**0.75**	0.01	0.04
**Sad**	0.00	0.14	0.71	**0.14**	0.02
**Happy**	0.05	0.29	0.57	0.09	**0.00**
**Average accuracy:**		**0.18 n.s.**			

Note. Confusion matrices for six methods-related variables. Rows are true emotion categories, and columns are brain-based predicted categories. Numbers are proportions (range 0-1). *: $p < .05$.
