# Supplementary material for: A Bayesian Model of Category-Specific Emotional Brain Responses
Source: PLoS Comput Biol. 2015 Apr 8;11(4):e1004066. doi: 10.1371/journal.pcbi.1004066 (PMC4390279; doi:10.1371/journal.pcbi.1004066)
Supplement: S3 Table — (PDF) [file pcbi.1004066.s004.pdf]

Supplementary Table S3. Emotion classification based on methodological variables

Overall intensity across zone (FDR q < .05 : p = 0.0022)

Region group	ang-dis	ang-fea	ang-hap	ang-sad	dis-fea	dis-hap	dis-sad	fea-hap	fea-sad	hap-sad
Cortex		+			++					
Basal Ganglia										
Cerebellum/Brainstem										
Thalamus								+	+	
Amygdala	+		++	+++	--			++	+++	
Hippocampus	+				--	-		+	++	

Non-negative matrix factorization "fingerprints:" different intensity profiles across regions (FDR q < .05 : p = .0188)

Set	ang-dis	ang-fea	ang-hap	ang-sad	dis-fea	dis-hap	dis-sad	fea-hap	fea-sad	hap-sad
Cortex	***		***	***	***		*	***	***	*
Basal Ganglia			*	*	*		*			
Cerebellum/Brainstem		***		**						
Thalamus					**	*		*	*	
Amygdala	*		**	***	**			***	***	
Hippocampus	**	*	**		***	*		**	***	*

Note. Significant pairwise differences between emotion categories in overall average intensity of region groups/ networks (top) and profiles across networks/regions within each group (bottom). Empty cells are non-significant. */ +/- P < .05. **/++/-- P < .01. ***/+++/--- P < .001.
