# Supplementary figures and images for: A Bayesian Model of Category-Specific Emotional Brain Responses
Source: PLoS Comput Biol. 2015 Apr 8;11(4):e1004066. doi: 10.1371/journal.pcbi.1004066 (PMC4390279; doi:10.1371/journal.pcbi.1004066)

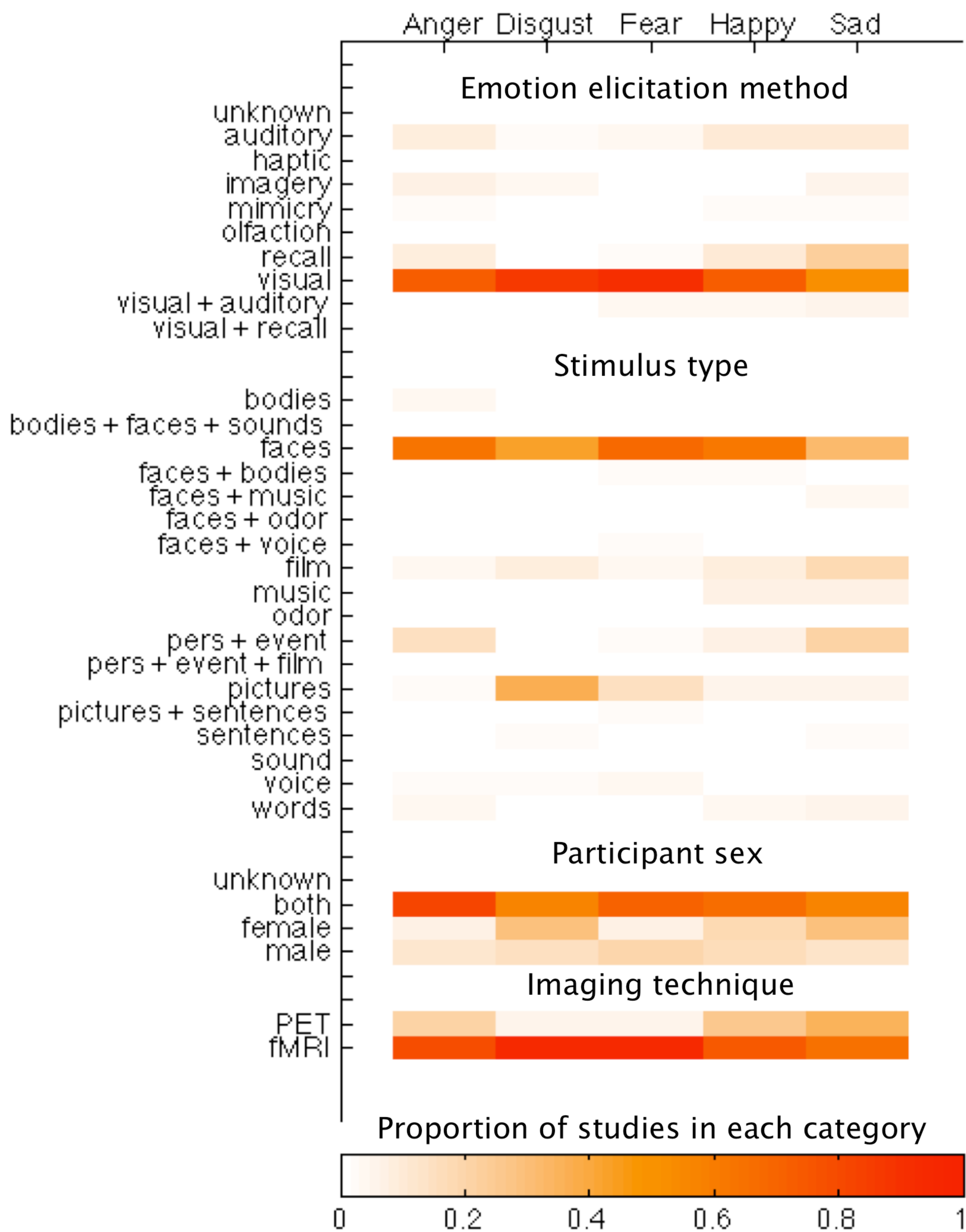

Supplement: S1 Fig — A) Map of relationships between emotion categories and methodological variables, including emotion elicitation method, stimulus type used, participant sex, and imaging technique. Colors represent proportions of studies in a given emotion category that involved each method variable; columns each sum to 1 (100% of studies) within each method variable. Ideally, stimulus category and other methodological variables would be evenly distributed across the five emotion categories (i.e., each row would be approximately evenly colored across emotion categories), although this is impractical in practice, and the distribution depends on how investigators have chosen to conduct studies. See S2 Table for additional information about classification of emotion type from these methodological variables. (PDF) [file pcbi.1004066.s005.pdf]

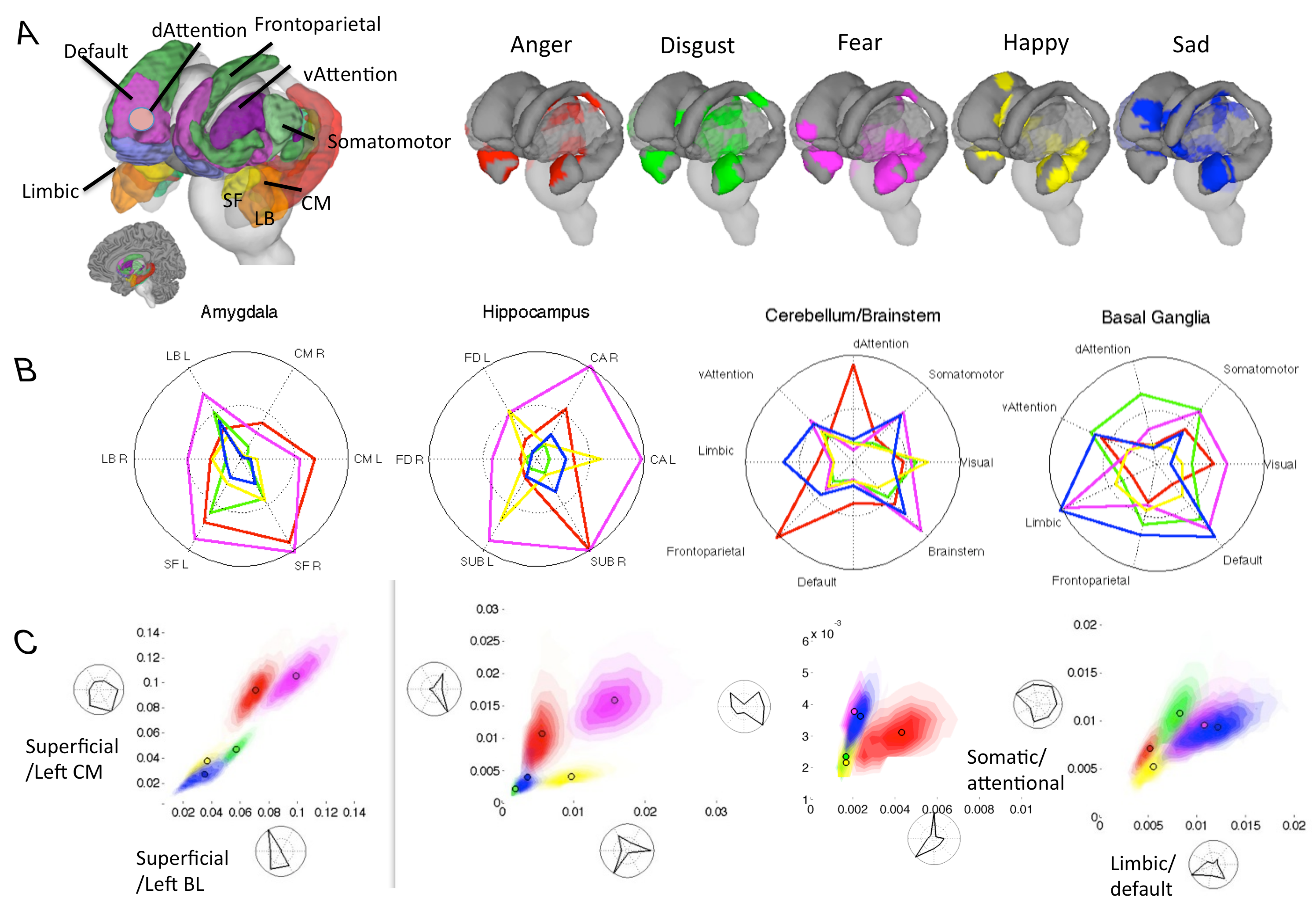

Supplement: S3 Fig — Subcortical zones and activation intensity profiles within each. A) Left: Basal ganglia regions of interest based on the Buckner Lab’s 1000-person resting state connectivity analyses (9–11), along with amygdala and parahippocampal/hippocampal regions of interest based on the probabilistic atlas of Amunts et al. (12) (see Main Text). Network labels follow the conventions used in the Buckner Lab papers. Right: Intensity maps for each emotion category displayed on surface images of the basal ganglia, amygdala, and hippocampus. B) Intensity profiles for each emotion category (colored lines, colors as in (A)) in subcortical zones. Values towards the perimeter of the circle indicate higher activation intensity. In the amygdala, LB: basolateral complex; CM: corticomedial division; SF: superficial division. L: left; R: right hemisphere. In the hippocampus: FD, dentate; CA, Cornu Ammonis; SUB, subicular zone. C) Intensity distribution for each emotion (colors) in the space of the first two factors from non-negative matrix factorization of the intensity profiles. The colors correspond to the emotion category labels in (A), and the colored areas show the 95% confidence region for the activation intensity profiles. (PDF) [file pcbi.1004066.s007.pdf]

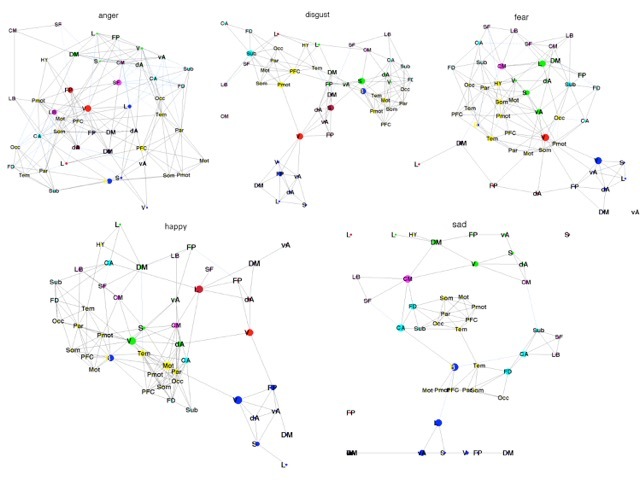

Supplement: S4 Fig — Connectivity graphs for each emotion category, as in Fig. 3, but with labels for all regions/networks. A) anger; B) disgust; C) fear; D) happy; E) sad. The layouts are force-directed graphs for each emotion category, based on the Fruchterman-Reingold spring algorithm. The nodes (circles) are regions or networks, color-coded by anatomical system. The edges (lines) reflect co-activation between pairs of regions or networks, assessed based on the joint distribution of activation intensity in the Bayesian model at P <. 05 corrected based on a permutation test. The size of each circle reflects its betweenness-centrality (ref), a measure of how strongly it connects disparate networks. Region labels are as in Figs. 2 and S3. Colors: Cortex, red; Basal ganglia, green; Cerebellum/brainstem, blue; Thalamus, yellow; Amygdala, magenta; Hippocampus, cyan/light blue. Network names follow the convention used in the Bucker Lab’s resting-state connectivity papers: V, visual network/zone (in cortex and connected regions in basal ganglia and cerebellum); dA, dorsal attention; vA, ventral attention; FP, fronto-parietal; S, somatosensory; DM, default mode; L, limbic. Thalamic regions are from the connectivity-based atlas of Behrens et al. (13). Tem, temporal; Som, somatosensory; Mot, motor; Pmot, premotor; Occ, occipital, PFC, prefrontal cortex connectivity. Other regions: Hy, hypothalamus; B (yellow letter on blue node), brainstem. (JPEG) [file pcbi.1004066.s008.jpeg]
