# Supplementary material for: A Bayesian Model of Category-Specific Emotional Brain Responses
Source: PLoS Comput Biol. 2015 Apr 8;11(4):e1004066. doi: 10.1371/journal.pcbi.1004066 (PMC4390279; doi:10.1371/journal.pcbi.1004066)
Supplement: S2 Fig — Intensity maps for each of the five emotion categories. Intensity maps reflect the distribution of study activation centers (Level 2 in the Bayesian model) across brain space. They are continuously valued across space, though they are sampled in voxels (2 x 2 x 2 mm), and the integral of the intensity map over any area of space reflects the expected number of study-level centers for that emotion category. Brighter colors indicate higher intensity, and the maps are thresholded at a value of 0.001 for display. (PDF) [file pcbi.1004066.s006.pdf]

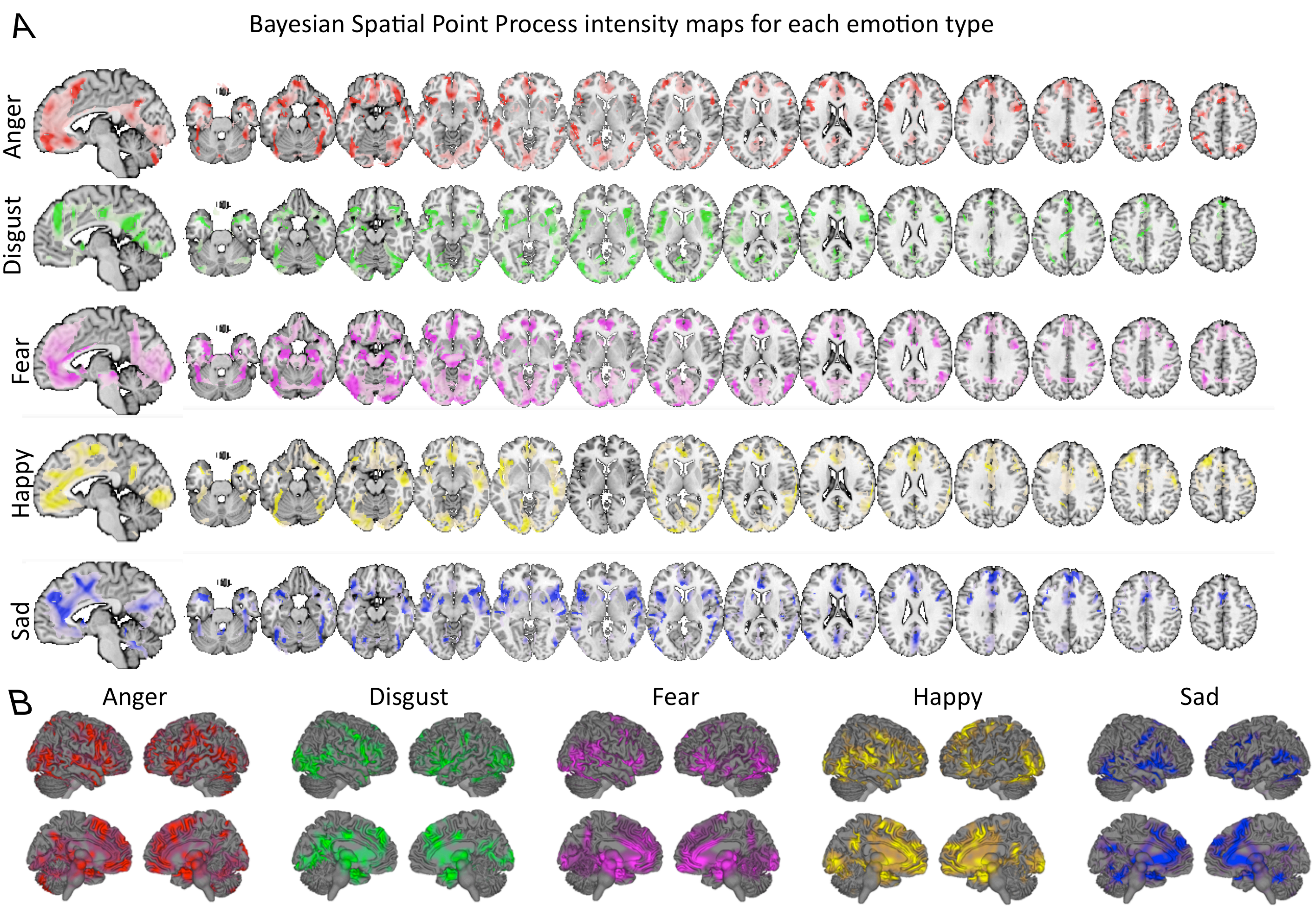
A
Bayesian Spatial Point Process intensity maps for each emotion type
Anger
Disgust
Fear
Happy
Sad
B
Anger
Disgust
Fear
Happy
Sad
