# Supplementary material for: A Bayesian Model of Category-Specific Emotional Brain Responses
Source: PLoS Comput Biol. 2015 Apr 8;11(4):e1004066. doi: 10.1371/journal.pcbi.1004066 (PMC4390279; doi:10.1371/journal.pcbi.1004066)

Supplementary Figure S5: Average within- and between-set correlations, and comparison with path length (global efficiency)

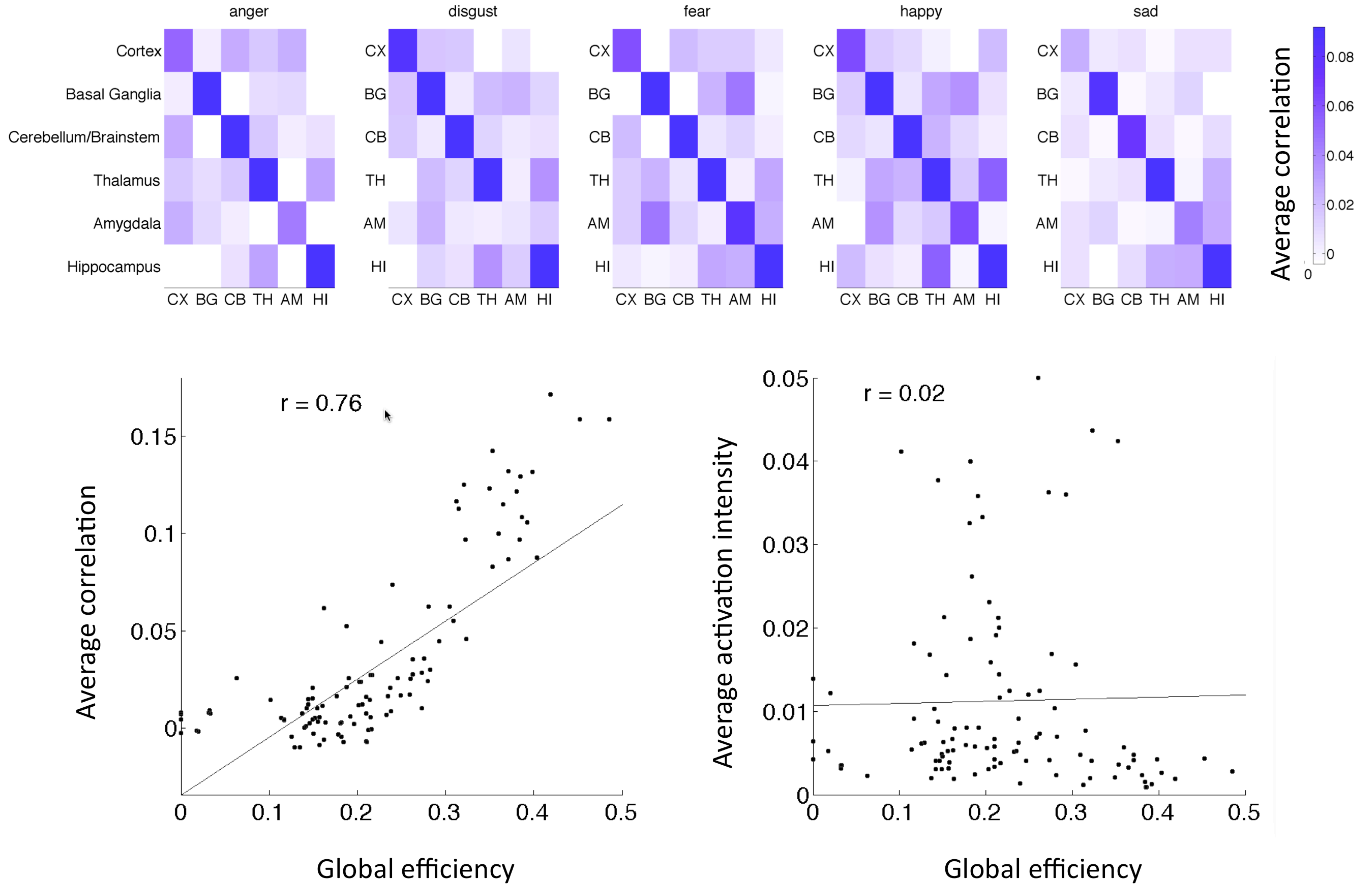

Supplement: S5 Fig — Average co-activation within and between each region/network grouping, for comparison to global network efficiency values based on path length in Fig. 3. Top: Average correlation in regional intensity across 10,000 MCMC samples in the Bayesian model. These correlations provide a measure of co-activation across disparate brain networks. The overall pattern is similar to Fig. 3; however, the average correlation does not reflect some of the structure captured in global efficiency and reflected in the graphs in Fig. 3. Bottom left: Average correlation is related to global efficiency across network groups and emotion categories (r = 0.76). Each point reflects an element of the matrices in the top panel. Bottom right: Global efficiency is unrelated to average activation intensity within the regions being correlated (r = 0.02), indicating that the efficiency metric used in the main manuscript provides information independent of the marginal activation intensity. (PDF) [file pcbi.1004066.s009.pdf]
